# The Flipped Classroom in Medical Education: Systematic Review and Meta-Analysis

**DOI:** 10.2196/60757

**Published:** 2025-08-13

**Authors:** Dragan Spaic, Zoran Bukumiric, Nina Rajovic, Ksenija Markovic, Marko Savic, Jelena Milin-Lazovic, Nikola Grubor, Nikola Milic, Dejana Stanisavljevic, Aleksa Despotovic, Dejan Bokonjic, Jelena Vladicic Masic, Valerija Janicijevic, Srdjan Masic, Natasa Milic

**Affiliations:** 1 Department of Primary Health Care and Public Health Faculty of Medicine University of East Sarajevo Foca Bosnia and Herzegovina; 2 Institute of Medical Statistics and Informatics University of Belgrade Faculty of Medicine Belgrade Serbia; 3 Department of Pediatrics Faculty of Medicine University of East Sarajevo Foca Bosnia and Herzegovina; 4 Department of Internal Medicine Faculty of Medicine University of East Sarajevo Foca Bosnia and Herzegovina; 5 University of Belgrade Faculty of Education Belgrade Serbia

**Keywords:** flipped classroom, medical education, meta-analysis

## Abstract

**Background:**

The concept of flipped classrooms (FCs) is gaining attention in medical education as it aligns with the digital age’s demand for more interactive and accessible learning experiences. By shifting the delivery of instructional content outside of the classroom, an FC allows students to engage with materials at their own pace, thereby maximizing in-class time for discussions, problem-solving, and other active learning activities.

**Objective:**

This study aimed to conduct a comprehensive meta-analysis to appraise the comparative effectiveness of FC instruction in contrast to traditional pedagogical modalities, with a particular focus on postepidemic analyses within specific subfields of medical education.

**Methods:**

The PubMed, Web of Science, and Scopus databases were systematically searched for studies comparing academic outcomes between the FC and traditional learning approaches in medical education. The primary outcome measures were knowledge assessment and students’ satisfaction. The standardized mean difference (SMD) was used as a measure of the overall effect, and subgroup analysis was performed according to the study design (randomized controlled trial [RCT] vs observational). The Cochran *Q* test and Baujat plots were used to estimate heterogeneity, coupled with *I*^2^. Highly influential studies were identified; sensitivity analyses and metaregression were performed.

**Results:**

In total, 141 studies were included in the systematic review; 127 (90.1%) studies with 21,171 participants were included in the meta-analysis of students’ knowledge assessment, of which 37 (29.1%) were RCTs. FCs had significantly better outcomes than the traditional method in knowledge test scores in both observational studies and RCTs (SMD 0.90, 95% CI 0.59-1.20, *P*<.001 and SMD 0.93, 95% CI 0.65-1.22, *P*<.001, respectively). There was substantial heterogeneity among included studies (*I*^2^=95.2%, τ^2^=1.614; *P*<.001). The funnel plot showed high asymmetry with significant small study effects (*P*<.001). However, the effect estimate remained robust to the exclusion of highly influential studies in the sensitivity analysis. In total, 27 (21.3%) studies with a total of 5842 participants reported students’ satisfaction. Higher student satisfaction scores for FCs were demonstrated in contrast to control groups (SMD 0.82, 95% CI 0.45-1.19; *P*<.001). There was substantial heterogeneity among the included studies (*I*^2^=97.8%, τ^2^=0.913; *P*<.001) but no evidence for publication bias, and no studies were found to be influential.

**Conclusions:**

The FC method is associated with better knowledge achievement and greater student satisfaction than the traditional approach in medical education, paving the way for its broader integration into medical school curricula. However, it is essential to consider various factors, such as the availability of resources, faculty readiness, and student preferences when implementing any new educational approach. This study holds promise for advancing medical education by exploring innovative teaching methodologies that leverage technology to enhance learning outcomes.

## Introduction

The rapid advancement of emerging technologies has prompted a reassessment of prevailing educational methodologies [1-3]. The wide accessibility of modern information and communication (IC) technologies has fundamentally altered the means of information retrieval, collaborative endeavors, and communication channels. These technologies have facilitated a novel learning paradigm wherein students enjoy and benefit from immediate and constant access to educational resources. While conventional pedagogical approaches have historically served to disseminate information broadly, they may exhibit shortcomings in cultivating solution-oriented competencies and practical knowledge application among students [4]. Consequently, there has been a rising scholarly interest in the flipped classroom (FC) model, also referred to as “flipped learning,” which represents a pedagogical framework that disrupts conventional learning modalities by integrating traditional face-to-face instruction with online educational platforms [5]. It involves the delivery of foundational content outside of traditional class hours, often via digital mediums. Conversely, activities traditionally designated as homework, such as problem-solving exercises and application-oriented tasks, are relocated to the classroom environment [2]. This instructional approach typically necessitates students’ independent engagement with prerecorded lectures or online materials before in-person instructional sessions, which are structured around student-centered activities aimed at fostering the application of previously acquired knowledge [6].

Moreover, the inconsistency inherent in traditional formative assessment practices and the limited avenues for communication between educators and students present challenges in addressing the entirety of the learning continuum. However, the FC model may offer solutions to these challenges associated with foundational learning and motivational impediments [7,8]. As traditional paradigms of medical education demand considerable temporal and resource allocations from both instructors and learners, the assessment of the potential impact of an FC and its practical use within the current medical education curricula has become paramount in enhancing educational efficacy [9-12]. Nonetheless, entrenched inertia within large academic institutions often impedes the adoption of innovative pedagogical approaches, notwithstanding meta-analytic findings substantiating the efficacy of FCs in health-related education [13,14]. This resistance to change is partly attributable to historical gaps in training directed toward students and educators in leveraging IC technologies within instructional contexts [15]. Successful navigation of technologically enriched educational milieux within the medical domain necessitates the acquisition of IC competencies, compounded by challenges uniquely relevant to medical education.

A recent systematic review and meta-analysis assessing the effects of an FC on students’ learning demonstrated a large variance across various disciplines and academic levels, with medicine being among those scientific fields having the lowest effect [16]. These findings further support the role of assessing the possible moderators and confounds [16] to explain the true impact of the FC model on advanced learning in a particular academic field. However, among the 46 identified meta-analyses, just a few examined student performances in an FC compared to the traditional learning environment for health care professionals (in a pre−COVID-19 pandemic setting), and none with a specific focus on higher medical education, excluding related fields, such as dentistry, pharmacy, nursing, and others. In addition, the postpandemic landscape warrants a reevaluation of the prospects for FC implementation, especially in medical fields. In response to the pressing circumstances during the COVID-19 pandemic, medical faculties have undergone adaptations to operate within pandemic-induced restrictions, bolstering capacities for digital engagement through intensive training initiatives targeting students and faculties on IC technologies use within instructional frameworks [17]. Thus, in this study, we aimed to conduct a comprehensive systematic review and meta-analysis to appraise the comparative effectiveness of FC instruction in contrast to traditional pedagogical modalities, with a particular focus on postepidemic analyses within specific subfields of medical education. This approach aims to address gaps identified in the published literature and highlight the relevance of an FC in evolving medical education contexts.

## Methods

The systematic review was conducted per the PRISMA (Preferred Reporting Items for Systematic Reviews and Meta-Analyses) guidelines of Observational Studies in Epidemiology [18,19]. The search strategy was developed by a biostatistician and informatician with expertise in conducting systematic reviews and meta-analyses and experience using IC technologies as educational tools (NM and SM).

### Search Strategy

The PubMed, Web of Science, and Scopus databases were searched until October 10, 2024, for studies containing the following search terms: “(flip* OR invert*) AND (classroom OR learn* OR instruction* OR course*) AND medic*.”

### Eligibility Criteria

Rayyan, a web-based technology platform for conducting systematic reviews, was used to store publications, delete duplicates, record the reviewer’s decisions, and help resolve conflicts. After removing duplicates, 2 reviewers independently screened potentially relevant titles and abstracts. The papers were equally distributed among reviewer pairs (D Spaic and NR; KM and MS; JM-L and NG; and Nikola Milic and AD). Discrepancies during the screening process were resolved through discussion, either with the involvement of a third reviewer (D Stanisavljevic, DB, JVM, VJ, SM, and Natasa Milic) or by reaching a consensus among the reviewers. Before proceeding with the full-text screening process, studies underwent inclusion based on eligibility criteria and exclusion if the title and abstract lacked sufficient information. The following inclusion criteria were used: (1) studies that compared FCs with any other learning method, (2) studies focusing on medical students, and (3) original articles.

Articles containing any of the following were excluded: (1) studies exclusively evaluating the efficacy of the FC approach (ie, studies without control group) or studies that did not evaluate the FC approach; (2) studies that did not report outcomes of interest (ie, knowledge assessment or students’ satisfaction); (3) studies that included health care professionals or students of disciplines other than medicine; (4) studies not written in the English language; and (5) publications that were book chapters, reviews, case reports, editorials, letters to the editor, conference abstracts, and theses. The full-text papers were equally distributed among reviewer pairs (D Spaic and NR; KM and MS; JM-L and NG; and Nikola Milic and AD), and each reviewer independently screened the article’s text. In the case of disagreements, a third reviewer (D Stanisavljevic, DB, JVM, VJ, SM, and Natasa Milic) independently evaluated the articles and made the final decision regarding their inclusion or exclusion.

### Data Extraction

Two independent reviewers extracted the following data: authors, publication year, country of origin, study population, study design (randomized controlled trial [RCT] or non-RCT design and whether baseline testing was conducted), duration of intervention, sample size, age, and sex. The following relevant outcomes were extracted: student knowledge assessment and student satisfaction with used learning approach.

### Individual Effect Size Pooling, Study Heterogeneity, and Risk of Bias

The primary outcome measures were knowledge assessment and students’ satisfaction, presented as means with SD. Students’ satisfaction reported as a proportion of students satisfied with the applied teaching approach was also assessed. The standardized mean difference (SMD) was used as a measure of the overall effect, and subgroup analysis was performed according to study design (RCTs vs observational). Random-effects models via the Der Simonian-Laird method were used to pool individual trial results, examine differences between the means of the FC and control group in units of SDs, and estimate SMDs. GetData Graph Digitizer (version 2.26.0.20 [20]) was used to read knowledge test scores and students’ satisfaction levels when figures but no tables were available in the original article. In cases where means and/or SDs were not reported, the median was used to approximate the arithmetic mean, and IQR/1.35 was used to approximate the SD. If SEs were reported, the SD was calculated as SD = SE × √n. In cases of reporting ranges, the SD was estimated as (maximum−minimum)/4. Cochran *Q* test and Baujat plots were used to estimate heterogeneity. Furthermore, we have reported *I*^2^ heterogeneity measures considering the known limitations of Cochran *Q* tests. According to the Cochrane Handbook, *I*^2^<30%, *I*^2^=30%-60%, and *I*^2^>60 correspond to low, moderate, and high heterogeneity, respectively.

A separate forest plot was presented for each analysis, showing the SMD (box), 95% CI (lines), and weight (size of box) for each publication. We used the diamond symbol to represent the overall effect size (ES). Prediction interval, based on t-distribution, was also estimated and presented in addition to SMDs. Furthermore, SMDs were interpreted using Cohen *d* ES, and common language ESs were calculated to better communicate the differences between the FC and control groups. A *P* value of <.05 was considered statistically significant for all analyses. Data visualization and statistical analysis were done using R *meta* and *metafor* packages (version 4.0.0; R Foundation for Statistical Computing).

### Influence Analysis

A separate analysis was conducted to identify highly influential studies and quantify their impact by evaluating the standardized residuals, difference in fits (DFFITS), Cook distance, leave-one-out 𝜏^2^ (LOO-𝜏^2^), and Cochrane *Q* metrics for differential heterogeneity assessment. Influence analysis was performed using the *dmetar* package.

### Sensitivity Analysis

Sensitivity analysis was conducted to examine the effects of (1) the exclusion of highly influential studies and (2) the replacement of knowledge test values in studies where multiple tests were performed.

### Publication Bias

Publication bias was assessed by funnel plots. An Egger test was performed and reported alongside the funnel plots to quantify the amount of asymmetry. The trim-and-fill method was used to identify and impute hypothetically missing studies to correct for funnel plot asymmetry, providing an adjusted estimate of the effect.

### Risk of Bias

Two reviewers independently evaluated the potential for bias in each RCT and the overall quality of the collected evidence using the Risk of Bias 2 tool, a component of the Cochrane Collaboration’s framework for assessing bias in randomized trials. The Risk of Bias 2 tool examines multiple domains of bias, including those related to the randomization process, deviations from intended interventions, missing outcome data, outcome measurement, and the selection of reported results. For observational studies, the reviewers independently assessed the risk of bias within each study using an adapted version of the Newcastle-Ottawa tool and the guidelines outlined by the Grading of Recommendations, Assessment, Development, and Evaluations Working Group.

### Metaregression

Univariate and multivariate metaregression analyses were performed for final knowledge assessment as an outcome measure. Due to a lot of missing data on predictors within studies, we performed predictor selection (1) based on the frequency of reporting in a minimum of 100 studies and (2) by performing forward stepwise selection on a subset of studies with complete cases, optimizing for the Akaike Information Criterion.

## Results

### Study Selection

**Figure 1:**
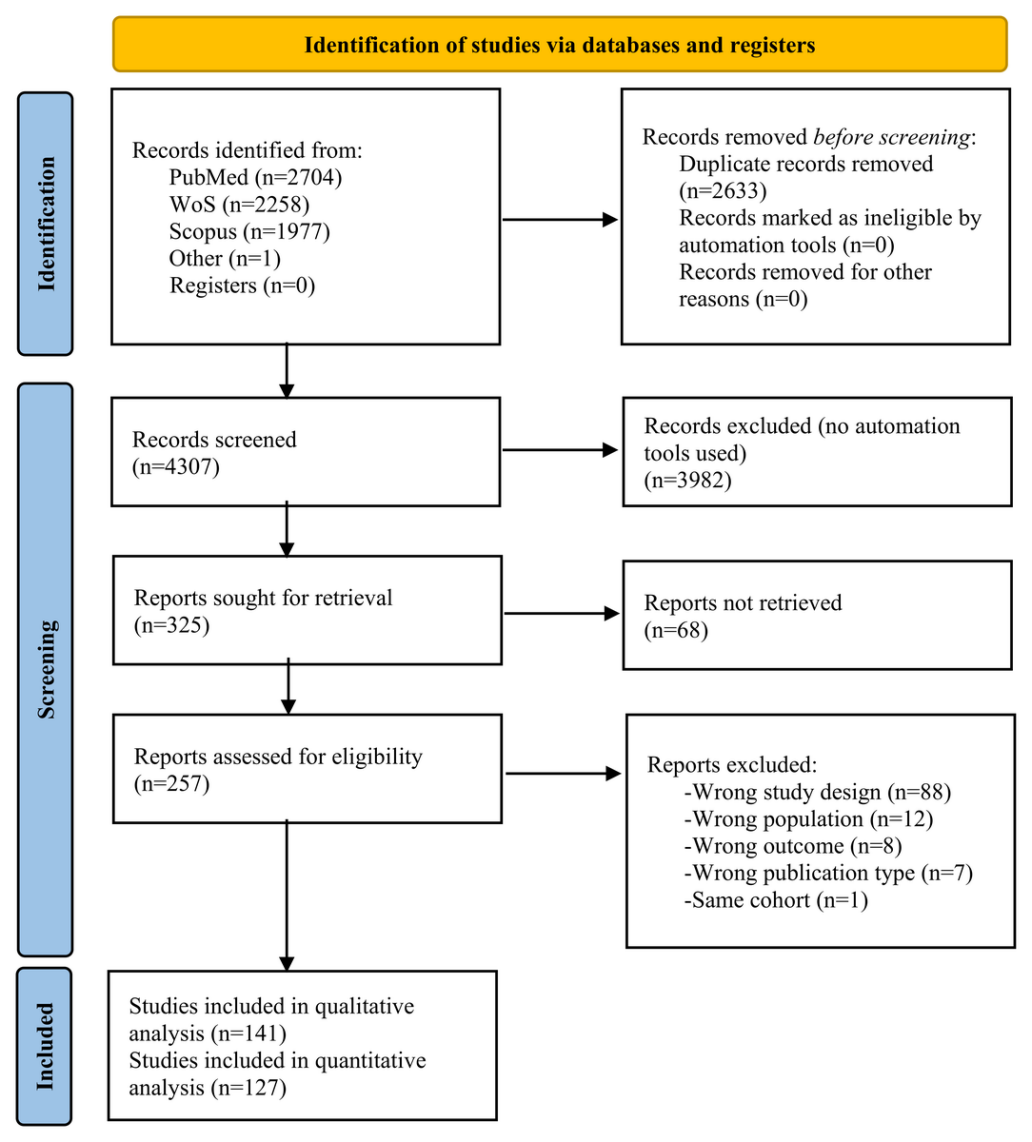
Flowchart of study selection process. WoS: Web of Science.

A total of 6940 potentially eligible publications were found. After removing duplicates, 4307 abstracts were screened for eligibility. Of the 325 publications sought for retrieval, 257 full-text articles were reviewed and 141 were selected for inclusion in the systematic review [21-161]. A flowchart illustrating this selection process is presented in Figure 1.

### Study Characteristics

Among the 141 studies in the systematic review, 69 (48.9%) were conducted in the United States and China. Studies were published between 2013 and 2024, with sample sizes ranging from 18 to 1016 participants. The study groups’ ages ranged from a minimum average age of 18.9 years to 38.5 years. In 3 (2.1%) studies, all participants were female. Of the 141 studies, 111 (78.7%) included undergraduate students, while 29 (20.6%) involved postgraduate students. More than half of the studies (78/141, 55.3%) focused on clinical subjects, 45 (31.9%) on basic science subjects, and 9 (6.4%) on public health. Overall, 104 (73.8%) were observational studies, and 37 (26.2%) were RCTs. A baseline knowledge assessment was performed in 44 (31.2%) studies, while 91 (64.5%) studies included only a final knowledge assessment. Detailed breakdowns of all eligible studies included in the systematic review are presented in Table S1 in Multimedia Appendix 1 [21-161].

### Knowledge Assessment Between the FC and Control Group

In total, 127 studies with 21,171 participants (FC: n=9865; control: n=11,306) reported final knowledge scores. The meta-analysis results identified significant differences in final knowledge scores between the FC and control groups (SMD 0.90, 95% CI 0.68-1.13; *P*<.001), thus favoring an FC (Table 1). However, there was substantial heterogeneity among included studies (*I*^2^=95.2%, τ^2^=1.6136; *P*<.001; Figure S1 in Multimedia Appendix 1) and the funnel plot showed high asymmetry (Figure S1 in Multimedia Appendix 1) with significant small study effects (*P*<.001). After applying the trim-and-fill method, the adjusted ES was attenuated (SMD 0.30, 95% CI 0.002-0.59; *P*=.049; Table 2).

**Figure 2:**
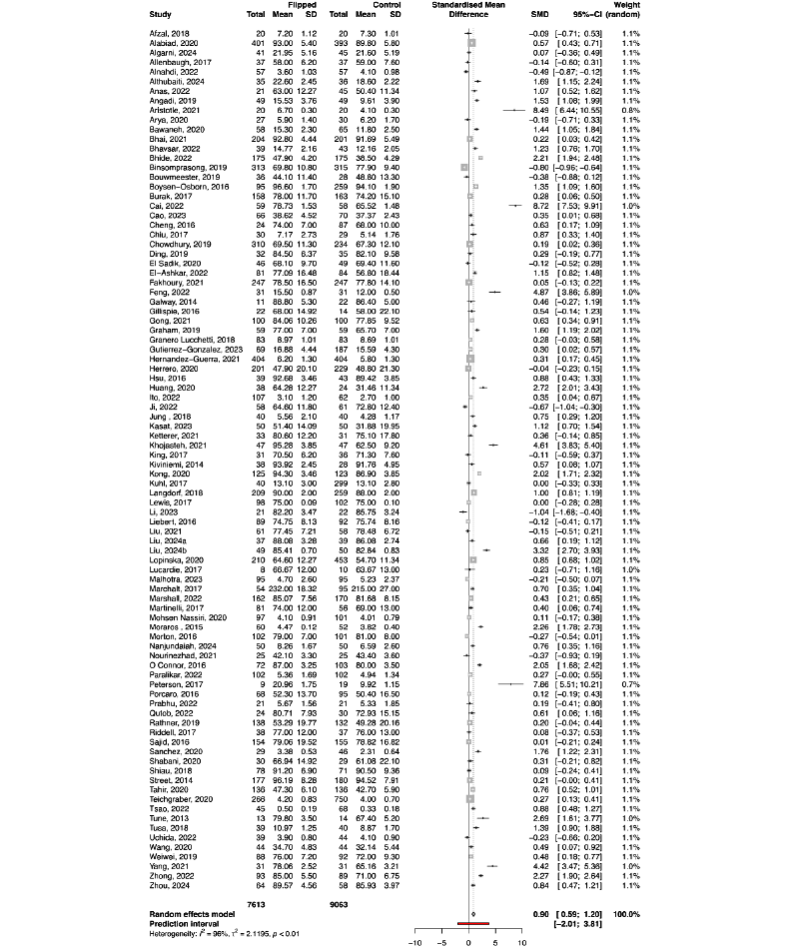
Forest plot comparing final knowledge scores between the flipped classroom and control group in observational studies. Forest plot of final test score-01 in observational studies.

**Figure 3:**
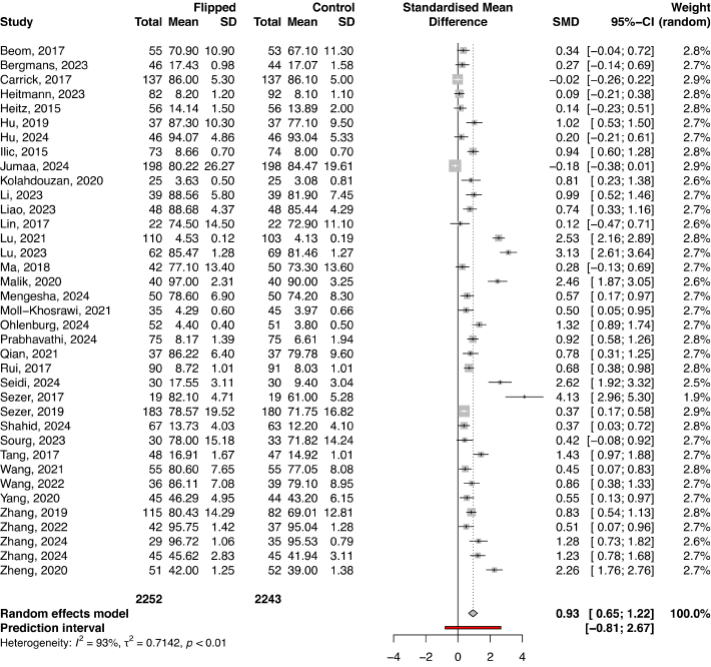
Forest plot comparing final knowledge scores between the flipped classroom and control group in randomized controlled trials (RCTs). Forest plot of final test scores in RCTs.

The observational study design was used in 90 studies with a total of 16,676 participants (FC: n=7613; control: n=9063), while the RCT design was used in 37 studies with a total number of 4495 participants (FC: n=2252; control: n=2243). The final knowledge scores were higher in an FC in contrast to the control group in both observational studies and RCTs (SMD 0.90, 95% CI 0.59-1.20; *P*<.001 and SMD 0.93, 95% CI 0.65-1.22; *P*<.001, respectively; Figures 2 and 3). In both observational and RCT studies, there was a substantial heterogeneity among the included studies (*I*^2^=95.6%, τ^2^=2.1195, *P*<.001 and *I*^2^=93.2%, τ^2^=0.7142, *P*<.001, respectively). Regarding the assessment of risk of bias in the RCTs included in the meta-analysis, there were some concerns about bias in most of the included studies (Figure S2 in Multimedia Appendix 1). For observational studies, there was an overall moderate risk of bias, with the quality of included studies ranging from 4 to 9, the highest rating being 9. Additional subgroup analyses of studies reporting knowledge scores based on study level, study subject, course duration, and differences among instructors reveal the same large heterogeneity among the included studies (Table S2 in Multimedia Appendix 1).

The SMD for final knowledge scores in the overall meta-analysis, as well as in both observational studies and RCTs, indicated a large ES (≥0.8), with the FC group outperforming approximately 74% of individuals in the control group. Additional details on the common language ESs are provided in Table 1.

In the overall meta-analysis of the final knowledge score, 5 studies were found to be highly influential; however, the exclusion of these studies had no significant effect on the pooled effect estimate. The robustness of the effect estimate was also assessed in sensitivity analysis by rerunning the meta-analysis with outcomes substituted with alternatives in studies where they were available. The effect estimate remained robust to these changes (Table 2; Figure S3 in Multimedia Appendix 1).

**Table 1:** The standardized mean difference (SMD), Cohen effect size, and common language effect size (CLES) for final knowledge score and student satisfaction between the flipped classroom (FC) and control group.

Analysis			SMD (95% CI)		Effect size^a^		CLES (95% CI)
**Final knowledge score between the FC and control group**							
	All	0.90 (0.68-1.13)		Large		74 (68-79)	
	Observational	0.90 (0.59-1.20)		Large		74 (66-80)	
	RCTs^b^	0.93 (0.65-1.22)		Large		74 (68-81)	
**Final knowledge score in studies reporting baseline knowledge test**							
	All	0.97 (0.53-1.40)		Large		75 (65-84)	
	Observational	0.99 (0.22-1.76)		Large		76 (56-89)	
	RCTs	0.97 (0.50-1.44)		Large		75 (64-85)	
**Student satisfaction assessment**							
	All	0.82 (0.45-1.19)		Large		72 (62-80)	
	Observational	0.87 (0.39-1.35)		Large		73 (61-83)	
	RCTs	0.69 (0.17-1.22)		Medium		69 (55-81)	

^a^Cohen *d* standardized effect size.

^b^RCT: randomized controlled trial.

**Table 2:** Meta-analysis of studies reporting knowledge scores (overall effect, influence, sensitivity, and trim-and-fill analysis).

Analysis	SMD^a^ (95% CI)	95% prediction interval	*I*^2^ (95% CI)
Overall	0.90 (0.68-1.13)	−1.62 to 3.43	95.2 (94.6-95.6)
Influential study removed^b^	0.71 (0.55-0.86)	−0.98 to 2.40	94.3 (93.7-94.9)
Sensitivity analysis	0.84 (0.61-1.07)	−1.75 to 3.43	95.3 (94.8-95.8)
Trim and fill	0.30 (0.002-0.59)	−3.44 to 4.04	96.8 (96.5-97.0)

^a^SMD: standardized mean difference.

^b^Aristotle et al [31], 2021; Cai et al [45], 2022; Feng et al [57], 2022; Khojasteh et al [81], 2021; and Peterson et al [118], 2017.

### Knowledge Assessment in Studies Reporting a Baseline Knowledge Score

**Figure 4:**
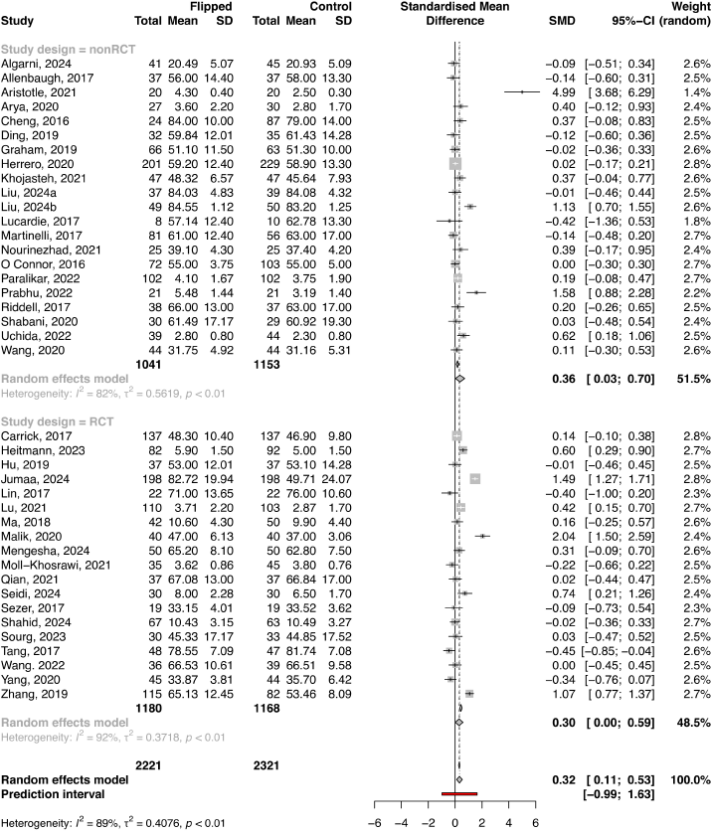
Forest plot for baseline knowledge scores between the flipped classroom and control group according to study design. Forest plot of baseline test scores.

In total, 40 studies with a total of 4542 cases (FC: n=2221; control: n=2321) implemented baseline knowledge assessment at study entry. The results of the meta-analysis presented differences in knowledge scores between the FC and control group at baseline (SMD 0.32, 95% CI 0.11-0.53; *P*=.003; Figure 4). The subgroup analysis revealed a significant difference in baseline knowledge between the FC and control group in observational studies (SMD 0.36, 95% CI 0.03-0.70), while in RCTs, there were no substantial differences (SMD 0.30, 95% CI 0.00-0.59; Figure 4).

**Figure 5:**
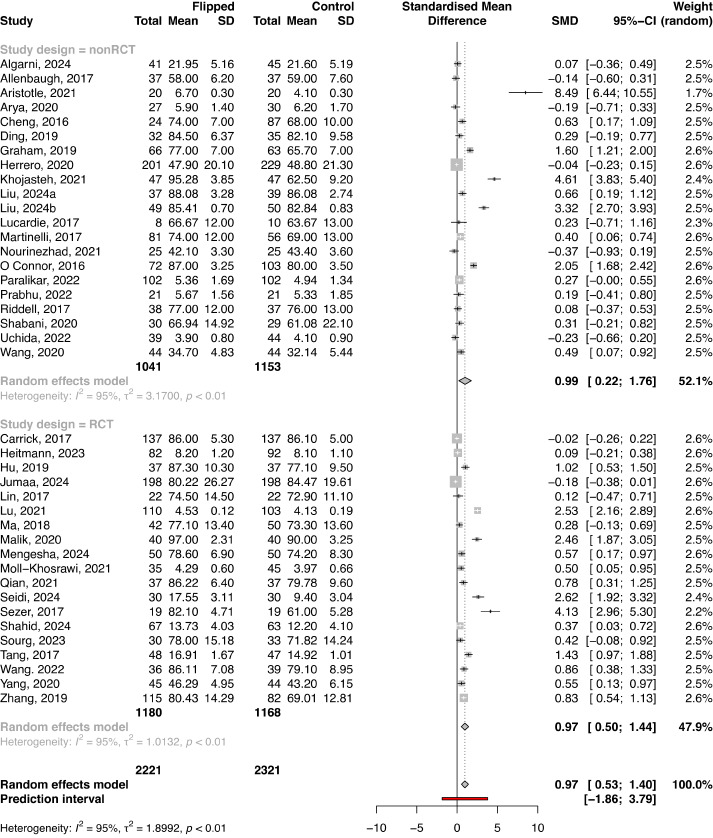
Forest plot comparing final knowledge scores between the flipped classroom and control group in studies reporting baseline knowledge test scores according to study design. Forest plot of final test scores in studies reporting baseline test.

The final knowledge scores in studies reporting baseline knowledge assessment were higher in an FC in contrast to the control group overall (SMD 0.97. 95% CI 0.53-1.40; *P*<.001; Figure 5), as well as in both observational studies and RCTs (SMD 0.99, 95% CI 0.22-1.76 and SMD 0.97, 95% CI 0.50-1.44, respectively; Figure 5). The SMD in the overall meta-analysis, as well as in both observational studies and RCTs, indicated a large ES (≥0.8), with the FC group outperforming approximately 75% of individuals in the control group in the overall analysis (Table 1).

Substantial heterogeneity was identified among included studies in both baseline and final analyses (*I*^2^=88.7%, τ^2^=0.41, *P*<.001 and *I*^2^=94.9%, τ^2^=1.90, *P*<.001, respectively; Figure S4 in Multimedia Appendix 1). The funnel plot found no evidence for publication bias in the assessment of baseline knowledge (*P*=.98; Figure S4 in Multimedia Appendix 1), while publication bias was found for the final knowledge assessment (*P*<.001; Figure S4 in Multimedia Appendix 1). The results of the trim-and-fill analysis presented an unchanged ES for the assessment of baseline knowledge, while an attenuated ES was present for the final knowledge assessment (Table 3).

Among studies that reported baseline knowledge scores, 2 studies were detected as influential in the assessment of baseline knowledge (Table 3; Figure S5 in Multimedia Appendix 1), and 2 highly influential studies were identified in the final knowledge assessment (Table 3; Figure S6 in Multimedia Appendix 1). However, sensitivity analysis presented no significant effect of these studies on the pooled effect estimates in both analyses.

**Table 3:** Meta-analysis of studies reporting baseline and final knowledge score (overall effect, influence, and trim-and-fill analysis).

Analysis		SMD^a^ (95% CI)	95% prediction interval		*I*^2^ (95% CI)
**Baseline knowledge score as outcome measure**					
	Overall	0.32 (0.11-0.53)	−0.99 to 1.63		88.7 (85.5-91.1)
	Influential study removed^b^	0.22 (0.07-0.37)	−0.64 to 1.08		85.5 (81.0-88.9)
	Trim and fill	0.32 (0.11-0.53)	−0.99 to 1.63		88.7 (85.5-91.1)
**Final knowledge score as outcome measure (in studies reporting baseline knowledge test)**					
	Overall	0.97 (0.53-1.40)		−1.86 to 3.79	94.9 (93.8-95.8)
	Influential study removed^c^	0.73 (0.42-1.04)		−1.20 to 2.67	93.8 (92.4-95.0)
	Trim and fill	0.64 (0.04-1.24)		−3.41 to 4.70	95.7 (94.8-96.4)

^a^SMD: standardized mean difference.

^b^Aristotle et al [31], 2021; Malik et al [103], 2020.

^c^Aristotle et al [31], 2021; Khojasteh et al [81], 2021.

### Student Satisfaction Assessment

**Figure 6:**
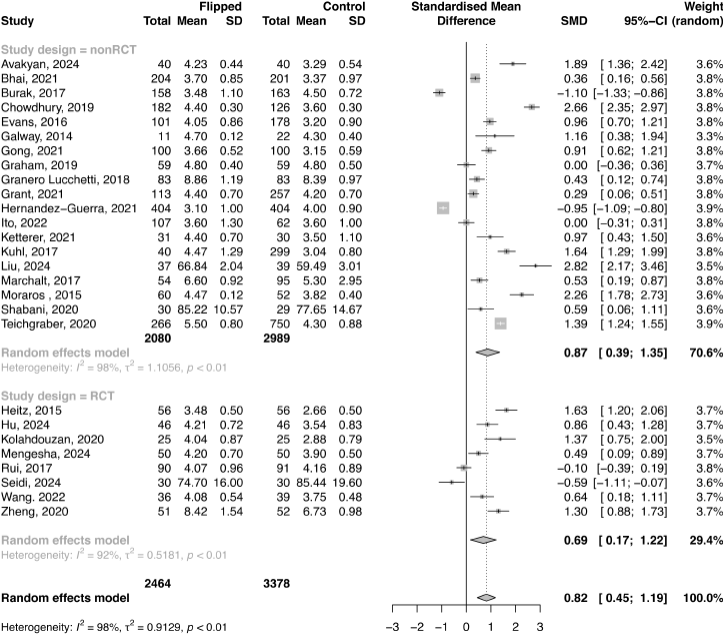
Forest plot comparing students’ satisfaction scores between the flipped classroom and control group according to study design. Forest plot of satisfaction scores.

A total of 27 studies with 5842 participants (FC: n=2464; control: n=3378) reported students’ satisfaction scores. The results of the meta-analysis overall effect showed higher students’ satisfaction scores for an FC in contrast to the control group (SMD 0.82, 95% CI 0.45-1.19; *P*<.001; Figure 6). There was substantial heterogeneity among the included studies (*I*^2^=97.8%, τ^2^=0.91, *P*<.001; Figure S7 in Multimedia Appendix 1) and borderline significance for publication bias (*P*=.051; Figure S7 in Multimedia Appendix 1), and no studies were found to be influential (Figure S8 in Multimedia Appendix 1). However, the results of the trim-and-fill analysis presented an attenuated ES (SMD 0.07, 95% CI −0.40 to 0.54; *P*=.76). The results of the subgroup meta-analysis showed significant differences in final knowledge scores between the FC and control group in both observational studies and RCTs; however, a lower ES was demonstrated in RCTs (SMD 0.87, 95% CI 0.39-1.35 and SMD 0.69, 95% CI 0.17-1.22, respectively; Figure 6).

The SMD in the overall analysis and in the analysis of observational studies indicated a large ES (≥0.8), with the FC group outperforming approximately 72% of individuals in the control group in the overall analysis. The SMD in the analysis of RCTs indicated a medium ES (0.69), with the FC group outperforming approximately 69% of individuals in the control group (Table 1).

**Figure 7:**
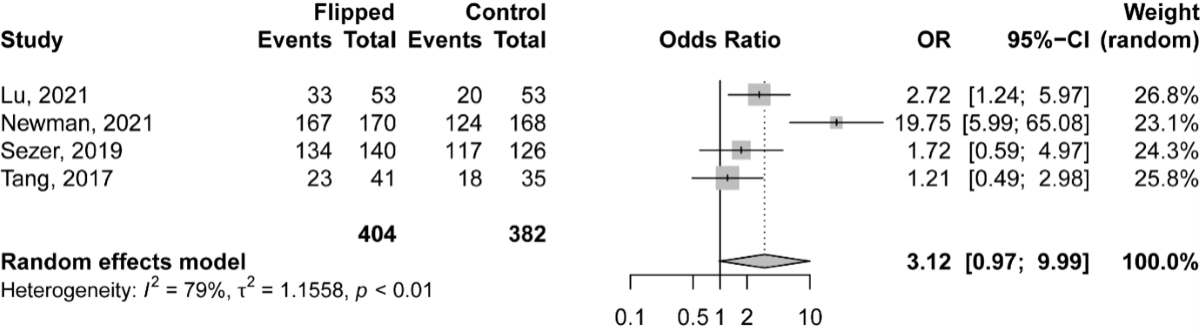
Forest plot comparing the proportion of students satisfied with the teaching approach between the flipped classroom and control group.

A total of 4 studies with 786 cases (FC: n=404; control: n=382) reported a proportion of students satisfied with the teaching approach. The results of the meta-analysis showed borderline differences in the proportion of satisfied students between the FC and control group (OR 3.1206, 95% CI 0.97-9.99; *P*=.06; Figure 7). There was substantial heterogeneity among the included studies (*I*^2^=79.2%, τ^2^=1.156, *P*<.001).

### Metaregression for Final Knowledge Test Scores as the Outcome Measure

Metaregression (Table 4) showed a significant correlation (*P*=.01) between the course students have undertaken and ES (basic science subjects having a larger ES) but no significant relationship between study design (RCT or observational), knowledge assessment reporting (baseline/final vs final), having same versus different instructors in compared groups, study level (undergraduate vs postgraduate), duration (semester vs block), students’ age, sex, period (before, during, or after the COVID-19 pandemic), and the outcome (*P*>.05 for all analysis).

Due to a lot of missing data on some predictors within studies, for multivariate modeling, we used only predictors that are reported in a minimal 100 studies; that is, study design (RCT/observational), knowledge assessment (baseline/final vs final), study level (undergraduate vs. postgraduate), duration (semester vs block), subject (clinic/public health vs basic), and period (before, during, or after the COVID-19 pandemic). The results of the multimodel inference coefficients (Table 5) and predictor importance analysis (Figure S9 in Multimedia Appendix 1) confirmed that the most important predictor of FC success is the specificity of the course students have undertaken, indicating that basic science subjects have a larger ES.

**Table 4:** Univariate metaregression for final knowledge test scores as the outcome measure.

Variables		Studies, n	Coefficient (95% CI)	*P* value
Study design (RCT^a^/observational)		127	0.069 (−0.428 to 0.566)	.79
Knowledge assessment (baseline/final vs final)		123	0.076 (−0.419 to 0.573)	.76
Instructors (same vs different in compared groups)		41	0.091 (−0.991 to 1.173)	.87
Study level (undergraduate vs. postgraduate)		127	−0.003 (−0.569 to 0.563)	.99
Duration (semester vs block)		105	−0.025 (−0.530 to 0.481)	.92
Age (y)		44	−0.061 (−0.185 to 0.062)	.33
Subject (clinic/public health vs basic)		122	−0.609 (−1.091 to −0.126)	.01
Sex (female; %)		44	0.001 (−0.018 to 0.020)	.92
**Period**				
	Before the COVID-19 pandemic (reference)	88	—^b^	—
	During the COVID-19 pandemic	19	0.105 (−0.566 to 0.775)	.78
	After the COVID-19 pandemic	11	0.037 (−0.810 to 0.883)	.88

^a^RCT: randomized controlled trial.

^b^Not applicable.

**Table 5:** Multimodel inference coefficients for final knowledge scores as outcome measure.

Variables			Coefficient		*P* value
Study design (RCT^a^/observational)			0.040		.88
Knowledge assessment (baseline/final vs final)			0.178		.59
Study level (undergraduate vs postgraduate)			−0.007		.98
Duration (semester vs block)			−0.232		.47
Subject (clinic/public health vs basic)			−0.900		.008
**Period**					
	Before the COVID-19 pandemic (reference)	—^b^		—	
	During the COVID-19 pandemic	0.184		.62	
	After the COVID-19 pandemic	−0.118		.78	

^a^RCT: randomized controlled trial.

^b^Not applicable.

## Discussion

In this study, we conducted a comprehensive meta-analysis to appraise the comparative effectiveness of FC instruction in contrast to traditional pedagogical modalities in the context of medical education. The meta-analysis includes 127 studies, of which 37 are RCTs, thus enabling the assessment of a global effect measure of the FC strategy. Overall, we found a substantial positive effect (ES=0.90) of the FC approach. The pooled ES is comparably more pronounced than other fields (mathematics: ES=0.38; science: ES=0.50; science, technology, engineering, and mathematics: ES=0.90), suggesting that medical training is particularly amenable to the FC pedagogical strategy [162].

Most previously published meta-analyses included studies across various disciplines and academic levels, which limited the generalizability of their results to medical education. In addition, many studies from the medical educational context were observational in design, providing lower-quality evidence regarding the true effect of the FC model. In our study, we focused exclusively on studies related to medical education, excluding other disciplines and related fields such as dentistry, pharmacy, and nursing. We expanded the search strategy to include studies from after the COVID-19 pandemic period and presented ESs for the FC model in comparison to traditional learning methods, using high-quality study designs such as RCTs. It is important to note that the COVID-19 pandemic presented both novel challenges and opportunities for integrating technological innovations into medical pedagogy [163]. In our study, standardized ESs between RCT and observational studies were similar across medical disciplines and favored FC methods (observational: SMD 0.90, 95% CI 0.59-1.20, *P*<.001 and RCT: SMD 0.93, 95% CI 0.65-1.22, *P*<.001, respectively). However, between-study heterogeneity remained high irrespective of the type of study. This heterogeneity is likely a consequence of varied methodologies in FC application, partially because there is yet to be a universally accepted pedagogical implementation of the framework. Therefore, a distribution of ESs exists among the study population with differing success rates. Some inconsistently used sources of added heterogeneity could be preclass assessments, differing amounts of technology used, multiple types of handout material used, the use of group-based methods, the use of problem-based learning, the use of simulation exercises, and differences in outcome assessment. For example, the inconsistent use of preclass assessment (eg, quizzing) to maintain student engagement and enforce preparedness introduces added heterogeneity. Preclass assessment is a recognized mediator associated with larger ESs [162].

In addition, studies differed in the amount, quality, and type of technologies involved, some providing only prerecorded lecture material and others incorporating student lecture annotation with review, text-based handout material, or multiple types of student activities [164]. At other times, the scope of the applied FC model was significantly different, with some studies using complete curriculum redesigns and extensive lecture material having to be adapted [97]. In contrast, others (especially in the clinical setting) explored one-time learning opportunities tackling a single clinical concept [28,70]. The FC effect is highest when both quizzes and exercises (problem-based learning) are used together. However, the optimal mediator choice must be tailored to the curriculum at hand [162,165]. Studies also used group-based learning, project assignments, team-based assessment, and other ways to foster engagement [165].

Conceptually, the FC model should strive to replace instructor-driven passive learning with student-centered active learning. Acquiring clinical knowledge and skills requires active learning with an emphasis on problem-based learning, simulation sessions, clinical cases, and group-based learning—all activities where the FC approach is advantageous [166-168]. Given that active learning has proven benefits for knowledge acquisition and application, the question then becomes of identifying the salient features of the FC approach [162,169]. Material availability, self-paced learning, personalized teaching, and student-driven activities are thought to predict better learning outcomes [110,162,165]. The critical question is maintaining student engagement to use class time most effectively. Quizzing students in an FC model at the beginning of class is an external motivator for them to engage with the material before class, suggesting that some of the model’s effectiveness can be attributed to factors enforcing motivation. This preparatory assessment drives students to study in anticipation of the quizzes, leveraging external motivation to ensure they come to class equipped with the necessary knowledge at the cost of increased workload [165,166,170]. Increased performance also reflects increased workload and opportunity to practice. In one study, the number of classes required was doubled in the treatment arm [28]. However, these increases in workload might only scale if applied to part of the school curriculum.

There is also the looming question of outcome measurement. Final assessments could suffer from “teaching to the test” and not reflect any real-world knowledge application [169,171]. For example, FC approaches could increase test performance. However, if the application is not evaluated (through simulations and otherwise), these differences may not reflect the sought outcome—creating high-quality medical professionals.

Differences in learning objectives are the most striking delimiter between basic science and clinical subjects. Clinical subjects should teach students clinical skills in addition to clinical reasoning, compared to basic sciences, which do not have the added logistical hurdle of requiring simulation exams. Consequently, the ES of the flipped model is impacted by the discrepancy between the learning approach and assessment method (skill demonstration or test performance). For example, it would be impractical to assign students to make slides for their preclass assessment if the objective was to master the act of physical examination of the eye [23,93,169]. Curricula that provided students with examination stations and where students had access to simulation-based learning fared better in assessment [60]. In our study, subjects providing basic science skills presented a larger effect of the FC model in contrast to clinical subjects and the public health field. However, in some studies, problem-based learning was an excellent introductory approach to teach clinical skills, and studies that used case-based group discussions have noted increased scores in clinical case assessment [28,70], augmenting the goal of attaining relevant clinical skills [169].

Student engagement can be fostered through group-based discussions to diffuse student responsibility and grow independence. Problem-based learning and simulation can be used when clinical skills are the learning objective to develop clinical reasoning that can be generalized to the real-world setting. Recorded lecture material is vital as its availability makes it easy for self-paced learning and review for both physical examination and medical procedures [164]. However, the successful implementation of the FC approach requires careful planning, along with both technical and pedagogical support for instructors. Clearly defined learning objectives and aligning classroom activities with those objectives are critical for effective implementation [162]. One of the main challenges for instructors is the development of interactive content and structuring of lectures for independent student learning. These tasks demand additional time, technical resources, and a certain level of digital literacy from the teaching staff. Students may also face difficulties. Traditional laboratories and clinical practice offer direct sensory engagement and spontaneous learning opportunities through unforeseen situations—experiences that are difficult to replicate in a digital format. The absence of such practical, hands-on learning can weaken the connection between theoretical knowledge and real-world application. In addition, unequal access to digital resources due to unreliable internet connections or lack of appropriate equipment can exacerbate existing educational inequalities among students. This is why institutional efforts are also needed to ensure that students have equitable access to all necessary educational resources.

Student evaluations are commonly used to assess class and instructor quality. Indeed, student feedback showed significant satisfaction with FC learning models compared to traditional methods [67,156,159,172,173]. In both clinical and preclinical contexts, students expressed high levels of satisfaction, with flipped learning being the majority preferred strategy. Therefore, assessing student perception and its impact on teaching course satisfaction should be weighed when considering teaching reform. Importantly, current research demonstrates that student satisfaction may explain a minimal portion of learning variation [174]. There are concerns that student evaluations exhibit a systematic bias, making them poor markers of teaching effectiveness [175,176]. Some reports have found that despite the greater effectiveness of the flipped model, student satisfaction remained the same or even decreased [177,178]. This inconsistent effect of satisfaction could indicate that, for some learning objectives, the workload needed to achieve a better outcome might not align with students’ perceptions. The level of satisfaction could be related to factors irrelevant to the set objective [174,176,179]. The FC model tends to increase immediate workload in exchange for delayed but better outcomes. Further curricula optimization would entail differentiating excellent and bad workloads, which impacts learning and student satisfaction [170,180,181]. Allowing students to learn at their own pace and providing more opportunities for hands-on and engaging activities during class seem like good pedagogical tools [60,62,109,162]. Finally, the effectiveness of incorporating active learning seems to have the greatest impact on the overall effects of an FC [163].

### Limitations

The findings of this study should be interpreted with caution because of the high methodological diversity related to study design and outcome measures, as well as statistical heterogeneity. Incomplete reporting was evident in the assessed literature; some studies may have reported only major findings, making it difficult to evaluate their methodology or to reuse their data. Our meta-analysis did not include conference papers, dissertations, and papers on languages other than English, which may have introduced a selection bias.

### Conclusions

This study has the potential to advance medical education by exploring innovative, technology-enhanced teaching methodologies that improve learning outcomes. Its findings provide valuable insights for educators, curriculum developers, and policy makers regarding the efficacy of the FC model in medical education. The FC approach has been shown to promote better knowledge acquisition and higher student satisfaction compared to traditional methods, supporting its broader integration into medical school curricula, particularly for basic science subjects. However, successful implementation requires careful consideration of factors such as resource availability, faculty preparedness, and student preferences. To promote the FC model in low-resource settings, institutions should leverage free digital tools and open educational resources; prioritize low-cost, high-impact in-class strategies such as peer instruction and group discussions; and maximize existing infrastructure through community collaboration. In addition, future research should explore the integration of technology-assisted teaching tools, including artificial intelligence and adaptive learning platforms, to further enhance the effectiveness and scalability of FC methodologies in medical education.

## Appendix

Multimedia Appendix 1 Supplementary table and figures.

Multimedia Appendix 2 PRISMA checklist.

## Abbreviations

ES: effect size
FC: flipped classroom
IC: information and communication
PRISMA: Preferred Reporting Items for Systematic Reviews and Meta-Analyses
RCT: randomized controlled trial
SMD: standardized mean difference

## References

[1] Haleem A, Javaid M, Qadri MA, Suman R (2022). Understanding the role of digital technologies in education: a review. Sustain Oper Comput.

[2] Strelan P, Osborn A, Palmer E (2020). The flipped classroom: a meta-analysis of effects on student performance across disciplines and education levels. Educ Res Rev.

[3] Means B, Toyama Y, Murphy R, Baki M (2013). The effectiveness of online and blended learning: a meta-analysis of the empirical literature. Teach Coll Rec.

[4] Luscombe C, Montgomery J (2016). Exploring medical student learning in the large group teaching environment: examining current practice to inform curricular development. BMC Med Educ.

[5] Chen F, Lui AM, Martinelli SM (2017). A systematic review of the effectiveness of flipped classrooms in medical education. Med Educ.

[6] Prober CG, Heath C (2012). Lecture halls without lectures--a proposal for medical education. N Engl J Med.

[7] Akçayır G, Akçayır M (2018). The flipped classroom: a review of its advantages and challenges. Comput Educ.

[8] Abeysekera L, Dawson P (2014). Motivation and cognitive load in the flipped classroom: definition, rationale and a call for research. High Educ Res Dev.

[9] Pereira JA, Pleguezuelos E, Merí A, Molina-Ros A, Molina-Tomás MC, Masdeu C (2007). Effectiveness of using blended learning strategies for teaching and learning human anatomy. Med Educ.

[10] Morton CE, Saleh SN, Smith SF, Hemani A, Ameen A, Bennie TD, Toro-Troconis M (2016). Blended learning: how can we optimise undergraduate student engagement?. BMC Med Educ.

[11] Milic NM, Trajkovic GZ, Bukumiric ZM, Cirkovic A, Nikolic IM, Milin JS, Milic NV, Savic MD, Corac AM, Marinkovic JM, Stanisavljevic DM (2016). Improving education in medical statistics: implementing a blended learning model in the existing curriculum. PLoS One.

[12] Milic N, Masic S, Bjegovic-Mikanovic V, Trajkovic G, Marinkovic J, Milin-Lazovic J, Bukumiric Z, Savic M, Cirkovic A, Gajic M, Stanisavljevic D (2018). Blended learning is an effective strategy for acquiring competence in public health biostatistics. Int J Public Health.

[13] Chen KS, Monrouxe L, Lu YH, Jenq CC, Chang YJ, Chang YC, Chai PY (2018). Academic outcomes of flipped classroom learning: a meta-analysis. Med Educ.

[14] Hew KF, Lo CK (2018). Flipped classroom improves student learning in health professions education: a meta-analysis. BMC Med Educ.

[15] O'Doherty D, Dromey M, Lougheed J, Hannigan A, Last J, McGrath D (2018). Barriers and solutions to online learning in medical education - an integrative review. BMC Med Educ.

[16] Kapur M, Hattie J, Grossman I, Sinha T (2022). Fail, flip, fix, and feed – rethinking flipped learning: a review of meta-analyses and a subsequent meta-analysis. Front Educ.

[17] Divjak B, Rienties B, Iniesto F, Vondra P, Žižak M (2022). Flipped classrooms in higher education during the COVID-19 pandemic: findings and future research recommendations. Int J Educ Technol High Educ.

[18] Liberati A, Altman DG, Tetzlaff J, Mulrow C, Gøtzsche PC, Ioannidis JP, Clarke M, Devereaux PJ, Kleijnen J, Moher D (2009). The PRISMA statement for reporting systematic reviews and meta-analyses of studies that evaluate health care interventions: explanation and elaboration. PLoS Med.

[19] Stroup DF, Berlin JA, Morton SC, Olkin I, Williamson GD, Rennie D, Moher D, Becker BJ, Sipe TA, Thacker SB (2000). Meta-analysis of observational studies in epidemiology: a proposal for reporting. Meta-analysis Of Observational Studies in Epidemiology (MOOSE) group. JAMA.

[20] GetData Graph Digitizer homepage. GetData Graph Digitizer.

[21] Abali EE, Rashid H, Copeland HL, Calt M, DeMaio R, Patel J, Schild S, Phadtare S, Chai L, Ullo M (2020). Shaping perceptions of basic science education by utilizing real patient encounters. Med Sci Educ.

[22] Afzal S, Masroor I (2019). Flipped classroom model for teaching undergraduate students in radiology. J Coll Physicians Surg Pak.

[23] Alabiad CR, Moore KJ, Green DP, Kofoed M, Mechaber AJ, Karp CL (2020). The flipped classroom: an innovative approach to medical education in ophthalmology. J Acad Ophthalmol (2017).

[24] Algarni A (2024). Biomedical students' self-efficacy and academic performance by gender in a flipped learning haematology course. BMC Med Educ.

[25] Allenbaugh J, Spagnoletti C, Berlacher K (2019). Effects of a flipped classroom curriculum on inpatient cardiology resident education. J Grad Med Educ.

[26] Alnahdi M, Agha S, Khan MA, Almansour M (2022). The flipped classroom model: exploring the effect on the knowledge retention of medical students. J Ayub Med Coll Abbottabad.

[27] Althubaiti A, Althubaiti SM (2024). Flipping the online classroom to teach statistical data analysis software: a quasi-experimental study. SAGE Open.

[28] Anas S, Kyrou I, Rand-Weaver M, Karteris E (2022). The effect of online and in-person team-based learning (TBL) on undergraduate endocrinology teaching during COVID-19 pandemic. BMC Med Educ.

[29] Angadi NB, Kavi A, Shetty K, Hashilkar NK (2019). Effectiveness of flipped classroom as a teaching-learning method among undergraduate medical students - an interventional study. J Educ Health Promot.

[30] Arathi MS, Devi GD, Sharath K, Johnson WM, Bhandari A (2022). Effectiveness of flipped class room approach as a teaching methodology in anatomy for early clinical exposure modules for first-year medical students – an interventional study. Int J Anat Res.

[31] Aristotle S, Subramanian S, Jayakumar S (2021). Effectiveness of flipped classroom model in teaching histology for first-year MBBS students based on competency-based blended learning: an interventional study. J Educ Health Promot.

[32] Arya V, Gehlawat VK, Rana R, Kaushik J (2020). Flipped classroom versus traditional lecture in training undergraduates in pediatric epilepsy. J Family Med Prim Care.

[33] Avakyan EI, Taylor DC (2024). The effect of flipped learning on students' basic psychological needs and its association with self-esteem. BMC Med Educ.

[34] Bawaneh AK, Moumene AB (2020). Flipping the classroom for optimizing undergraduate students’ motivation and understanding of medical physics concepts. Eurasia J Math Sci Tech Ed.

[35] Belfi LM, Bartolotta RJ, Giambrone AE, Davi C, Min RJ (2015). "Flipping" the introductory clerkship in radiology: impact on medical student performance and perceptions. Acad Radiol.

[36] Beom JH, Kim JH, Chung HS, Kim SM, Ko DR, Cho J (2018). Flipped-classroom training in advanced cardiopulmonary life support. PLoS One.

[37] Bergmans E, Billington A, Thies KC (2023). From tradition to innovation: a comparison of the traditional 4-step approach versus a blended learning modification for technical skills teaching. Scand J Trauma Resusc Emerg Med.

[38] Bhai S, Poustinchian B (2021). The flipped classroom: a novel approach to physical examination skills for osteopathic medical students. J Osteopath Med.

[39] Bhavsar MH, Javia HN, Mehta SJ (2022). Flipped classroom versus traditional didactic classroom in medical teaching: a comparative study. Cureus.

[40] Bhide A, Singh S, Pujitha K, Vani P (2022). A study of impact of flipped classroom on student educational experience in comparison with didactic lecture in topics classified based on Bloom’s taxonomy. Biomedicine.

[41] Homsanit M (2019). Comparison of academic outcomes of epidemiology and biostatistics course between traditional lecture and flipped classroom blended approach in 2nd year medical students: a retrospective cohort study. Siriraj Med J.

[42] Bouwmeester RA, de Kleijn RA, van den Berg IE, ten Cate OT, van Rijen HV, Westerveld HE (2019). Flipping the medical classroom: effect on workload, interactivity, motivation and retention of knowledge. Comput Educ.

[43] Boysen-Osborn M, Anderson CL, Navarro R, Yanuck J, Strom S, McCoy CE, Youm J, Ypma-Wong MF, Langdorf MI (2016). Flipping the advanced cardiac life support classroom with team-based learning: comparison of cognitive testing performance for medical students at the University of California, Irvine, United States. J Educ Eval Health Prof.

[44] Burak KW, Raman M, Paget M, Busche K, Coderre S, McLaughlin K (2017). A mixed methods study on the effect of flipping the undergraduate medical classroom. Educ Sci.

[45] Cai L, Li YL, Hu XY, Li R (2022). Implementation of flipped classroom combined with case-based learning: a promising and effective teaching modality in undergraduate pathology education. Medicine (Baltimore).

[46] Cao YX, Xia SL, Zhu ZY, Zeng FR, Li HN, Zhang TT, Liu YJ (2023). Exploring lemology teaching with "internet plus" flipped classroom pedagogy. BMC Med Educ.

[47] Carrick FR, Abdulrahman M, Hankir A, Zayaruzny M, Najem K, Lungchukiet P, Edwards RA (2017). Randomized controlled study of a remote flipped classroom neuro-otology curriculum. Front Neurol.

[48] Cheng X, Ka Ho Lee K, Chang EY, Yang X (2017). The "flipped classroom" approach: stimulating positive learning attitudes and improving mastery of histology among medical students. Anat Sci Educ.

[49] Chick RC, Adams AM, Peace KM, Kemp Bohan PM, Schwantes IR, Clifton GT, Vicente D, Propper B, Newhook T, Grubbs EG, Bednarski BK, Vreeland TJ (2021). Using the flipped classroom model in surgical education: efficacy and trainee perception. J Surg Educ.

[50] Chiu HY, Kang YN, Wang WL, Huang HC, Wu CC, Hsu W, Tong YS, Wei PL (2018). The effectiveness of a simulation-based flipped classroom in the acquisition of laparoscopic suturing skills in medical students-a pilot study. J Surg Educ.

[51] Chowdhury TA, Khan H, Druce MR, Drake WM, Rajakariar R, Thuraisingham R, Dobbie H, Parvanta L, Chinegwundoh F, Almushatat A, Warrens A, Alstead EM (2019). Flipped learning: turning medical education upside down. Future Healthc J.

[52] Ding C, Li S, Chen B (2019). Effectiveness of flipped classroom combined with team-, case-, lecture- and evidence-based learning on ophthalmology teaching for eight-year program students. BMC Med Educ.

[53] El Sadik A, Al Abdulmonem W (2021). Improvement in student performance and perceptions through a flipped anatomy classroom: shifting from passive traditional to active blended learning. Anat Sci Educ.

[54] El-Ashkar A, Aboregela A, Abdelazim A, Metwally A (2022). Flipped classroom as a novel teaching tool for practical parasitology. Parasitologists United J.

[55] Evans KH, Thompson AC, O'Brien C, Bryant M, Basaviah P, Prober C, Popat RA (2016). An innovative blended preclinical curriculum in clinical epidemiology and biostatistics: impact on student satisfaction and performance. Acad Med.

[56] Fakhoury HM, A Fatoum H, Aldeiry MA, Alahmad H, Enabi J, Kayali S, Bawahab Y, Masuadi EM, Obeidat A, Lumsden CJ (2021). Flipping a biochemistry class within a medical curriculum: impacts on perception, engagement, and attainment. Biochem Mol Biol Educ.

[57] Feng Y, Zhao B, Zheng J, Fu Y, Jiang Y (2022). Online flipped classroom with team-based learning promoted learning activity in a clinical laboratory immunology class: response to the COVID-19 pandemic. BMC Med Educ.

[58] Galway LP, Corbett KK, Takaro TK, Tairyan K, Frank E (2014). A novel integration of online and flipped classroom instructional models in public health higher education. BMC Med Educ.

[59] Gillispie V (2016). Using the flipped classroom to bridge the gap to generation Y. Ochsner J.

[60] Gong J, Ruan M, Yang W, Peng M, Wang Z, Ouyang L, Yang G (2021). Application of blended learning approach in clinical skills to stimulate active learning attitudes and improve clinical practice among medical students. PeerJ.

[61] Graham KL, Cohen A, Reynolds EE, Huang GC (2019). Effect of a flipped classroom on knowledge acquisition and retention in an internal medicine residency program. J Grad Med Educ.

[62] Granero Lucchetti AL, Ezequiel OD, Oliveira IN, Moreira-Almeida A, Lucchetti G (2018). Using traditional or flipped classrooms to teach “geriatrics and gerontology”? Investigating the impact of active learning on medical students’ competences. Med Teach.

[63] Grant LL, Opperman MJ, Schiller B, Chastain J, Richardson JD, Eckel C, Plawecki MH (2021). Medical student engagement in a virtual learning environment positively correlates with course performance and satisfaction in psychiatry. Med Sci Educ.

[64] Gutiérrez-González R, Zamarron A, Royuela A, Rodriguez-Boto G (2023). Flipped classroom applied to neurosurgery in undergraduate medical education. BMC Med Educ.

[65] Heitmann H, Fischer E, Wagner P, Pötter D, Gartmeier M, Schmidt-Graf F (2023). Flipping the classroom in neurological bedside teaching: a prospective controlled study. BMC Med Educ.

[66] Heitz C, Prusakowski M, Willis G, Franck C (2015). Does the concept of the "flipped classroom" extend to the emergency medicine clinical clerkship?. West J Emerg Med.

[67] Hernández-Guerra M, Quintero E, Morales-Arráez DE, Carrillo-Pallarés A, Nicolás-Pérez D, Carrillo-Palau M, Gimeno-García A, González-Alayón C, Alarcón O, Otón-Nieto E, Díaz-Luis H, Hernández-Siverio N, Martín-Malagón A, Arteaga-González I, Bravo-Gutiérrez A, Lorenzo-Rocha M, Jordán-Balanza J, Sánchez-González JM, Barrera-Gómez M, Reid A, Marina N (2021). Comparison of flipped learning and traditional lecture method for teaching digestive system diseases in undergraduate medicine: a prospective non-randomized controlled trial. Med Teach.

[68] Herrero JI, Quiroga J (2020). Flipped classroom improves results in pathophysiology learning: results of a nonrandomized controlled study. Adv Physiol Educ.

[69] Hsu SD, Chen CJ, Chang WK, Hu YJ (2016). An investigation of the outcomes of PGY students' cognition of and persistent behavior in learning through the intervention of the flipped classroom in Taiwan. PLoS One.

[70] Hu X, Zhang H, Song Y, Wu C, Yang Q, Shi Z, Zhang X, Chen W (2019). Implementation of flipped classroom combined with problem-based learning: an approach to promote learning about hyperthyroidism in the endocrinology internship. BMC Med Educ.

[71] Hu B, Wang L, Wu J, Zhu L, Chen Z (2024). A combination of case-based learning with flipped classroom improved performance of medical students in nephrology bedside teaching. BMC Med Educ.

[72] Huang HL, Chou CP, Leu S, You HL, Tiao MM, Chen CH (2020). Effects of a quasi-experimental study of using flipped classroom approach to teach evidence-based medicine to medical technology students. BMC Med Educ.

[73] Ilic D, Nordin RB, Glasziou P, Tilson JK, Villanueva E (2015). A randomised controlled trial of a blended learning education intervention for teaching evidence-based medicine. BMC Med Educ.

[74] Ito A, Isohama Y, Watanabe K (2022). Comparison of flipped and traditional lecture-based classrooms for Kampo (traditional Japanese medicine) education in a medical school. Int J Educ Res Open.

[75] Jalali A, Jeong D, Sutherland S (2020). Implementing a competency-based approach to anatomy teaching: beginning with the end in mind. J Med Educ Curric Dev.

[76] Ji M, Luo Z, Feng D, Xiang Y, Xu J (2022). Short- and long-term influences of flipped classroom teaching in physiology course on medical students' learning effectiveness. Front Public Health.

[77] Jumaa MI, Hanafy SM, Farhat KH, Arafa MA, Farahat MF (2024). Student performance and perceptions of flipped classrooms and small group discussions as teaching tools in practical anatomy. Cureus.

[78] Jung H, An J, Park KH (2018). Analysis of satisfaction and academic achievement of medical students in a flipped class. Korean J Med Educ.

[79] Kasat P, Deshmukh V, Muthiyan GT, Sontakke B, Sorte SR, Tarnekar AM (2023). The role of the flipped classroom method in short-term and long-term retention among undergraduate medical students of anatomy. Cureus.

[80] Ketterer B, Childers JW, Arnold RM (2021). An innovative application of online learning for hospice education in medicine trainees. J Palliat Med.

[81] Khojasteh L, Hosseini SA, Nasiri E (2021). The impact of mediated learning on the academic writing performance of medical students in flipped and traditional classrooms: scaffolding techniques. Res Pract Technol Enhanc Learn.

[82] King AM, Mayer C, Barrie M, Greenberger S, Way DP (2018). Replacing lectures with small groups: the impact of flipping the residency conference day. West J Emerg Med.

[83] Kiviniemi MT (2014). Effects of a blended learning approach on student outcomes in a graduate-level public health course. BMC Med Educ.

[84] Kolahdouzan M, Mahmoudieh M, Rasti M, Omid A, Rostami A, Yamani N (2020). The effect of case-based teaching and flipped classroom methods in comparison with lecture method on learning and satisfaction of internship students in surgery. J Educ Health Promot.

[85] Kong F, Li Z, Su X, Zhuang W (2020). Assessment of a flipped classroom model based on microlectures in a medical molecular biology course. J Biol Educ.

[86] Kühl SJ, Toberer M, Keis O, Tolks D, Fischer MR, Kühl M (2017). Concept and benefits of the Inverted Classroom method for a competency-based biochemistry course in the pre-clinical stage of a human medicine course of studies. GMS J Med Educ.

[87] Langdorf MI, Anderson CL, Navarro RE, Strom S, McCoy CE, Youm J, Ypma-Wong MF (2018). Comparing the results of written testing for advanced cardiac life support teaching using team-based learning and the "flipped classroom" strategy. Cureus.

[88] Lewis CE, Chen DC, Relan A (2018). Implementation of a flipped classroom approach to promote active learning in the third-year surgery clerkship. Am J Surg.

[89] Li Y, Tang XF, Cheng H (2023). Application of a flipped classroom teaching model based on micro-videos in the standardized training of dermatological residents in China. Front Med (Lausanne).

[90] Li Y, Cao L, Zhang H, Pang W, Sun Y, Zhang Z (2023). Application of flipped classroom combined with case-based learning in Introduction to Environmental Health Science. Front Public Health.

[91] Liao W, He J, Yang C, Qi S, Chen G, Ding C (2023). Application of a new multi-element integrated teaching mode based on bite-sized teaching, flipped classroom, and MOOC in clinical teaching of obstetrics and gynaecology. BMC Med Educ.

[92] Liebert CA, Lin DT, Mazer LM, Bereknyei S, Lau JN (2016). Effectiveness of the Surgery Core Clerkship Flipped Classroom: a prospective cohort trial. Am J Surg.

[93] Lin Y, Zhu Y, Chen C, Wang W, Chen T, Li T, Li Y, Liu B, Lian Y, Lu L, Zou Y, Liu Y (2017). Facing the challenges in ophthalmology clerkship teaching: is flipped classroom the answer?. PLoS One.

[94] Liu S, Li Y, Wang X, Zhang X, Wang R (2021). Research on the effect of big data flipped classroom combined with scenario simulation teaching: based on clinical practice of medical students. Wireless Commun Mobile Comput.

[95] Liu K, Liu S, Ma Y, Jiang J, Liu Z, Wan Y (2024). Comparison of blended learning and traditional lecture method on learning outcomes in the evidence-based medicine course: a comparative study. BMC Med Educ.

[96] Liu Q, Tang XJ, Chen XK, Chen L (2024). Flipped classroom based on outcomes-based education improves student engagement and clinical analysis competence in undergraduates ophthalmology clerkship. Adv Med Educ Pract.

[97] Łopińska M, Gielecki JS, Żurada A (2022). Flipped spotters learning model: an innovative student activity-based strategy. A preparation tool for anatomy practical examinations in medical education. Anat Sci Educ.

[98] Lu RY, Yanovitch T, Enyedi L, Gandhi N, Gearinger M, de Alba Campomanes AG, Cavuoto KM, Gray M, Kemp PS, Silverstein E, Loh AR, Ding L, Cabrera MT (2021). The flipped-classroom approach to teaching horizontal strabismus in ophthalmology residency: a multicentered randomized controlled study. J AAPOS.

[99] Lu C, Xu J, Cao Y, Zhang Y, Liu X, Wen H, Yan Y, Wang J, Cai M, Zhu H (2023). Examining the effects of student-centered flipped classroom in physiology education. BMC Med Educ.

[100] Lucardie AT, Busari JO (2017). The flipped classroom as a pedagogical tool for leadership development in postgraduate medical education. Educ Sci.

[101] Ma X, Luo Y, Zhang L, Wang J, Liang Y, Yu H, Wu Y, Tan J, Cao M (2018). A trial and perceptions assessment of APP-based flipped classroom teaching model for medical students in learning immunology in China. Educ Sci.

[102] Malhotra AS, Bhagat A (2023). Flipped classroom for undergraduate medical students in India: are we ready for it?. Adv Physiol Educ.

[103] Malik SS, Yasmeen R, Khan RA (2020). A randomized controlled study to evaluate the effect of flipped classroom relative to a traditional demonstration method on learning of procedural skills in dermatology residents. J Pak Med Assoc.

[104] Marchalot A, Dureuil B, Veber B, Fellahi J, Hanouz JL, Dupont H, Lorne E, Gerard JL, Compère V (2018). Effectiveness of a blended learning course and flipped classroom in first year anaesthesia training. Anaesth Crit Care Pain Med.

[105] Marshall AM, Conroy ZE (2022). Effective and time-efficient implementation of a flipped-classroom in preclinical medical education. Med Sci Educ.

[106] Martinelli SM, Chen F, DiLorenzo AN, Mayer DC, Fairbanks S, Moran K, Ku C, Mitchell JD, Bowe EA, Royal KD, Hendrickse A, VanDyke K, Trawicki MC, Rankin D, Guldan GJ, Hand W, Gallagher C, Jacob Z, Zvara DA, McEvoy MD, Schell RM (2017). Results of a flipped classroom teaching approach in anesthesiology residents. J Grad Med Educ.

[107] Mengesha AK, Ayele HS, Misker MF, Beyna AT (2024). Assessing the effectiveness of flipped classroom teaching-learning method among undergraduate medical students at gondar university, college of medicine and health sciences: an interventional study. BMC Med Educ.

[108] Moll-Khosrawi P, Zöllner C, Cencin N, Schulte-Uentrop L (2021). Flipped learning enhances non-technical skill performance in simulation-based education: a randomised controlled trial. BMC Med Educ.

[109] Moraros J, Islam A, Yu S, Banow R, Schindelka B (2015). Flipping for success: evaluating the effectiveness of a novel teaching approach in a graduate level setting. BMC Med Educ.

[110] Morton DA, Colbert-Getz JM (2017). Measuring the impact of the flipped anatomy classroom: the importance of categorizing an assessment by Bloom's taxonomy. Anat Sci Educ.

[111] Moskowitz HS, Hsueh WD (2020). Integrative resident education curriculum to adapt to the modern otolaryngology trainee. Laryngoscope.

[112] Nanjundaiah K, Anuradha HV (2024). Comparison of flipped classroom versus traditional didactic lectures among medical students: a mixed method study. Natl J Clin Anat.

[113] Newman JR, Fink J, Clough LA, Johnston S (2021). Internal medicine clerkship ID curriculum flip: will they prefer to pre-learn?. Med Sci Educ.

[114] Nourinezhad S, Hadipourfard E, Bavali M (2021). The effect of audio-visual feedback on writing components and writing performance of medical university students in two different modes of instruction, flipped and traditional. Cogent Educ.

[115] O'Connor EE, Fried J, McNulty N, Shah P, Hogg JP, Lewis P, Zeffiro T, Agarwal V, Reddy S (2016). Flipping radiology education right side up. Acad Radiol.

[116] Ohlenburg H, Arnemann PH, Hessler M, Görlich D, Zarbock A, Friederichs H (2024). Flipped classroom: improved team performance during resuscitation training through interactive pre-course content - a cluster-randomised controlled study. BMC Med Educ.

[117] Paralikar S, Shah CJ, Joshi A, Kathrotia R (2022). Acquisition of higher-order cognitive skills (HOCS) using the flipped classroom model: a quasi-experimental study. Cureus.

[118] Peterson J, Louden DT, Gribben V, Blankenburg R (2017). Teaching residents clinical practice guidelines using a flipped classroom model. MedEdPORTAL.

[119] Pontius LN, Hooten J, Lesesky E, Rao C, Nicholas M, Bialas R, Liu B, Green CL, Atwater AR (2020). A comparison of knowledge acquisition and perceived efficacy of a traditional vs flipped classroom-based dermatology residency curriculum. Cutis.

[120] Porcaro PA, Jackson DE, McLaughlin PM, O’Malley CJ (2016). Curriculum design of a flipped classroom to enhance haematology learning. J Sci Educ Technol.

[121] Prabhavathi K, KalyaniPraba P, Rohini P, Selvi K, Saravanan A (2024). Flipped classroom as an effective educational tool in teaching physiology for first-year undergraduate medical students. J Educ Health Promot.

[122] Prabhu R, Prabhu G (2022). Flipped pedagogical approach in teaching skeletal muscle physiology for undergraduate medical students. Indian J Physiol Pharmacol.

[123] Qian Q, Yan Y, Xue F, Lin J, Zhang F, Zhao J (2021). Coronavirus disease 2019 (COVID-19) learning online: a flipped classroom based on micro-learning combined with case-based learning in undergraduate medical students. Adv Med Educ Pract.

[124] Qutob H (2022). Effect of flipped classroom approach in the teaching of a hematology course. PLoS One.

[125] Rathner JA, Schier MA (2020). The impact of flipped classroom andragogy on student assessment performance and perception of learning experience in two advanced physiology subjects. Adv Physiol Educ.

[126] Riddell J, Jhun P, Fung CC, Comes J, Sawtelle S, Tabatabai R, Joseph D, Shoenberger J, Chen E, Fee C, Swadron SP (2017). Does the flipped classroom improve learning in graduate medical education?. J Grad Med Educ.

[127] Rui Z, Lian-Rui X, Rong-Zheng Y, Jing Z, Xue-Hong W, Chuan Z (2017). Friend or foe? Flipped classroom for undergraduate electrocardiogram learning: a randomized controlled study. BMC Med Educ.

[128] Sajid MR, Laheji AF, Abothenain F, Salam Y, AlJayar D, Obeidat A (2016). Can blended learning and the flipped classroom improve student learning and satisfaction in Saudi Arabia?. Int J Med Educ.

[129] Sánchez JC, López-Zapata DF, Pinzón ÓA, García AM, Morales MD, Trujillo SE (2020). Effect of flipped classroom methodology on the student performance of gastrointestinal and renal physiology entrants and repeaters. BMC Med Educ.

[130] Seidi M, Ramezani-Aliakbari F, Doosti-Irani A (2024). Effectiveness of the flipped classroom method using clinical scenarios and educational technology versus subject-based lectures in a gastrointestinal physiology course for medical students. BMC Med Educ.

[131] Sezer B, Abay E (2018). Looking at the impact of the flipped classroom model in medical education. Scand J Educ Res.

[132] Sezer B, Elcin M (2020). Using traditional or flipped classrooms to teach “vascular access skill”: a pilot study to investigate the impact of the flipped classroom approach on students’ competencies. Soc Sci J.

[133] Shabani A, Mohammadi A, Mojtahedzadeh R, Hosseini A, Valadkhani S, Sistani A, Asadzandi S, Rashidi H (2020). Does the sequence of flipped and lecture-based classes affect the academic achievement and satisfaction of medical students?. J E-Learn Knowl Soc.

[134] Shahid U, Ahmer M, Anjum K, Mahmood N, Athar Z, Shahid A (2024). Medical education: comparison between flipped classroom and traditional classroom strategies in teaching human anatomy. JIIMC.

[135] Shiau S, Kahn LG, Platt J, Li C, Guzman JT, Kornhauser ZG, Keyes KM, Martins SS (2018). Evaluation of a flipped classroom approach to learning introductory epidemiology. BMC Med Educ.

[136] Shoemaker EZ, Johnson C, Hilty DM, Fung CC (2022). Flipping a single lecture in a survey course to active learning: do the benefits justify the costs?. J Technol Behav Sci.

[137] Smith E, Boscak A (2021). A virtual emergency: learning lessons from remote medical student education during the COVID-19 pandemic. Emerg Radiol.

[138] Sourg HA, Satti S, Ahmed N, Ahmed AB (2023). Impact of flipped classroom model in increasing the achievement for medical students. BMC Med Educ.

[139] Street SE, Gilliland KO, McNeil C, Royal K (2014). The flipped classroom improved medical student performance and satisfaction in a pre-clinical physiology course. Med Sci Educ.

[140] Tahir F, Hafiz B, Alnajjar T, Almehmadi B, Besharah B, Gari A, Katib Y (2020). Comparison of performance of medical students between two teaching modalities "flip the classroom" and traditional lectures: a single center educational interventional study. Pak J Med Sci.

[141] Tang F, Chen C, Zhu Y, Zuo C, Zhong Y, Wang N, Zhou L, Zou Y, Liang D (2017). Comparison between flipped classroom and lecture-based classroom in ophthalmology clerkship. Med Educ Online.

[142] Teichgräber U, Ingwersen M, Mentzel HJ, Aschenbach R, Neumann R, Franiel T, Herzog AB, Böttcher J, Pfeil A, Mensel B, Kühnel C, Freesmeyer M, Fischer MR, Zottmann J (2021). Impact of a heutagogical, multimedia-based teaching concept to promote self-determined, cooperative student learning in clinical radiology. Rofo.

[143] Nassiri Toosi M, Alizadeh M, Khodadadegi M, Armandei M, Ajalooian Z, Alikhani R (2021). Looking at the levels of Bloom's taxonomy in a flipped classroom utilizing study guide and interactive assignment for undergraduate medical students. Acta Med Iran.

[144] Tsao YP, Yeh WY, Hsu TF, Chow LH, Chen WC, Yang YY, Shulruf B, Chen CH, Cheng HM (2022). Implementing a flipped classroom model in an evidence-based medicine curriculum for pre-clinical medical students: evaluating learning effectiveness through prospective propensity score-matched cohorts. BMC Med Educ.

[145] Tune JD, Sturek M, Basile DP (2013). Flipped classroom model improves graduate student performance in cardiovascular, respiratory, and renal physiology. Adv Physiol Educ.

[146] Tusa N, Sointu E, Kastarinen H, Valtonen T, Kaasinen A, Hirsto L, Saarelainen M, Mäkitalo K, Mäntyselkä P (2018). Medical certificate education: controlled study between lectures and flipped classroom. BMC Med Educ.

[147] Uchida S, Shikino K, Ishizuka K, Yamauchi Y, Yanagita Y, Yokokawa D, Tsukamoto T, Noda K, Uehara T, Ikusaka M (2022). The flipped classroom is effective for medical students to improve deep tendon reflex examination skills: a mixed-method study. PLoS One.

[148] Veeramani R, Madhugiri VS, Chand P (2015). Perception of MBBS students to "flipped class room" approach in neuroanatomy module. Anat Cell Biol.

[149] Wang X, Li J, Wang C (2020). The effectiveness of flipped classroom on learning outcomes of medical statistics in a Chinese medical school. Biochem Mol Biol Educ.

[150] Wang T, Sun C, Mei YJ, Hou CY, Li ZJ (2021). Massive open online courses combined with flipped classroom: an approach to promote training of resident physicians in rheumatology. Int J Gen Med.

[151] Wang A, Xiao R, Zhang C, Yuan L, Lin N, Yan L, Wang Y, Yu J, Huang Q, Gan P, Xiong C, Xu Q, Liao H (2022). Effectiveness of a combined problem-based learning and flipped classroom teaching method in ophthalmic clinical skill training. BMC Med Educ.

[152] Weiwei B, Xiao J, Xiaoqiang Q, Yujia K, Chunzhen Z (2019). Practical exploration of the "rain classroom-flipped classroom" mode in basic chemistry course. Adv Soc Sci Educ Humanit Res.

[153] Yang C, Yang X, Yang H, Fan Y (2020). Flipped classroom combined with human anatomy web-based learning system shows promising effects in anatomy education. Medicine (Baltimore).

[154] Yang F, Lin W, Wang Y (2021). Flipped classroom combined with case-based learning is an effective teaching modality in nephrology clerkship. BMC Med Educ.

[155] Zhang XM, Yu JY, Yang Y, Feng CP, Lyu J, Xu SL (2019). A flipped classroom method based on a small private online course in physiology. Adv Physiol Educ.

[156] Zhang W, Gu J, Li F, Feng F, Chen H, Xing X, Liu L (2022). The effect of flipped classroom in multiple clinical skills training for clinical interns on Objective Structured Clinical Examinations (OSCE). Med Educ Online.

[157] Zhang W, Jiang M, Zhao W, Li S, Li F, Feng F, Wang Y, Li Y, Liu L (2024). Evaluation of the effectiveness of using flipped classroom in puncture skills teaching. BMC Med Educ.

[158] Zhang J, Chen H, Wang X, Huang X, Xie D (2024). Application of flipped classroom teaching method based on ADDIE concept in clinical teaching for neurology residents. BMC Med Educ.

[159] Zheng Z, Yuan S, Huang M, Liao J, Cai R, Zhan H, Yang Z, Xiong Y (2022). Flipped classroom approach used in the training of mass casualty triage for medical undergraduate students. Disaster Med Public Health Prep.

[160] Zhong J, Li Z, Hu X, Wang L, Chen Y (2022). Effectiveness comparison between blended learning of histology practical in flipped physical classrooms and flipped virtual classrooms for MBBS students. BMC Med Educ.

[161] Zhou X, Yu G, Li X, Zhang W, Nian X, Cui J, Wei X, Sun Y (2024). The application and influence of "small private online course" based on flipped classroom teaching model in the course of fundamental operations in surgery in China. Sci Rep.

[162] Hattie J (2023). Visible Learning: The Sequel: A Synthesis of Over 2,100 Meta-Analyses Relating to Achievement.

[163] Rose S (2020). Medical student education in the time of COVID-19. JAMA.

[164] Schmid RF, Bernard RM, Borokhovski E, Tamim RM, Abrami PC, Surkes MA, Wade CA, Woods J (2014). The effects of technology use in postsecondary education: a meta-analysis of classroom applications. Comput Educ.

[165] van Alten DC, Phielix C, Janssen J, Kester L (2019). Effects of flipping the classroom on learning outcomes and satisfaction: a meta-analysis. Educ Res Rev.

[166] Khanova J, Roth MT, Rodgers JE, McLaughlin JE (2015). Student experiences across multiple flipped courses in a single curriculum. Med Educ.

[167] Liebert CA, Mazer L, Bereknyei Merrell S, Lin DT, Lau JN (2016). Student perceptions of a simulation-based flipped classroom for the surgery clerkship: a mixed-methods study. Surgery.

[168] Ramnanan CJ, Pound LD (2017). Advances in medical education and practice: student perceptions of the flipped classroom. Adv Med Educ Pract.

[169] Mehta NB, Hull AL, Young JB, Stoller JK (2013). Just imagine: new paradigms for medical education. Acad Med.

[170] Marsh HW (2001). Distinguishing between good (useful) and bad workloads on students’ evaluations of teaching. Am Educ Res J.

[171] Oudbier J, Spaai G, Timmermans K, Boerboom T (2022). Enhancing the effectiveness of flipped classroom in health science education: a state-of-the-art review. BMC Med Educ.

[172] Gallardo NE, Caleya AM, Sánchez ME, Feijóo G (2022). Learning of paediatric dentistry with the flipped classroom model. Eur J Dent Educ.

[173] Smith EB, Boscak A, Friedman EM, Frand S, Deitte LA, Benefield T, Jordan S (2022). Radiology medical student education 2020: surveys of the alliance of medical student educators in radiology and medical students. Acad Radiol.

[174] Uttl B, White CA, Gonzalez DW (2017). Meta-analysis of faculty's teaching effectiveness: student evaluation of teaching ratings and student learning are not related. Stud Educ Eval.

[175] Stroebe W (2016). Why good teaching evaluations may reward bad teaching: on grade inflation and other unintended consequences of student evaluations. Perspect Psychol Sci.

[176] Boring A, Ottoboni K, Stark PB, Cetinkaya-Rundel M (2016). Student evaluations of teaching (mostly) do not measure teaching effectiveness. ScienceOpen Research.

[177] Della Ratta CB (2015). Flipping the classroom with team-based learning in undergraduate nursing education. Nurse Educ.

[178] Missildine K, Fountain R, Summers L, Gosselin K (2013). Flipping the classroom to improve student performance and satisfaction. J Nurs Educ.

[179] Spooren P, Brockx B, Mortelmans D (2013). On the validity of student evaluation of teaching: the state of the art. Rev Educ Res.

[180] Centra JA (2003). Will teachers receive higher student evaluations by giving higher grades and less course work?. Res High Educ.

[181] Greenwald AG, Gillmore GM (1997). No pain, no gain? The importance of measuring course workload in student ratings of instruction. J Educ Psychol.

