# Supplementary material for: The Flipped Classroom in Medical Education: Systematic Review and Meta-Analysis
Source: J Med Internet Res. 2025 Aug 13;27:e60757. doi: 10.2196/60757 (PMC12391848; doi:10.2196/60757)
Supplement: Multimedia Appendix 1 [file jmir_v27i1e60757_app1.docx]

**Table S1.** Characteristics of all eligible studies included in the systematic review.

| Study, year | Country | Population | Subject | Study design | Knowledge assessment | Duration | FC^a^/C^b^, n | Age (y; FC/C), mean (SD) | Women (FC/C), % |
| --- | --- | --- | --- | --- | --- | --- | --- | --- | --- |
| Abali et al [21], 2020 | United States | Undergraduate | Clinic | Non-RCT | Final | Semester | 333/333 | N/A^c^ | N/A |
| Afzal and Masroor [22], 2019 | Pakistan | Undergraduate | Clinic | Non-RCT | Final | Block | 20/20 | N/A | 55/60 |
| Alabiad et al [23], 2020 | United States | Undergraduate | Clinic | Non-RCT | Final | Block | 401/393 | N/A | N/A |
| Algarni [24], 2024 | Saudi Arabia | Undergraduate | Clinic | Non-RCT | Baseline and final | Semester | 41/45 | N/A | 46.3/46.7 |
| Allenbaugh et al [25], 2019 | United States | Postgraduate | N/A | Non-RCT | Baseline and final | Block | 37/37 | N/A | 51/46 |
| Alnahdi et al [26], 2022 | Saudi Arabia | Undergraduate | Clinic | Non-RCT | Final | Block | 57/57 | N/A | 45.6/59.6 |
| Althubaiti and Althubaiti [27], 2024 | Saudi Arabia | Undergraduate | Public Health | Non-RCT | Final | Block | 35/36 | N/A | 65.7/55.6 |
| Anas et al [28], 2022 | United Kingdom | Undergraduate | Clinic | Non-RCT | Final | Block | 21/45 | N/A | N/A |
| Angadi et al [29], 2019 | India | Undergraduate | Basic | Non-RCT | Final | Semester | 49/49 | N/A | N/A |
| Arathi et al [30], 2022 | India | Undergraduate | Basic | Non-RCT | Baseline and final | Block | N/A | N/A | N/A |
| Aristotle et al [31], 2021 | India | Undergraduate | Basic | Non-RCT | Baseline and final | Semester | 20/20 | N/A | N/A |
| Arya et al [32], 2020 | India | Undergraduate | Clinic | Non-RCT | Baseline and final | Block | 27/30 | N/A | N/A |
| Avakyan and Taylor [33], 2024 | United Arab Emirates | Undergraduate | Basic | Non-RCT | Final | Semester | 40/40 | 21.68 (1.25)/ 21.68 (1.25) | 60/60 |
| Bawaneh and Moumene [34], 2020 | Saudi Arabia | Undergraduate | Basic | Non-RCT | Final | N/A | 58/65 | N/A | 100/100 |
| Belfi et al [35], 2015 | United States | Undergraduate | Clinic | Non-RCT | Baseline and final | Block | N/A | N/A | N/A |
| Beom et al [36], 2018 | Korea | Undergraduate | Clinic | RCT | Final | Block | 55/53 | 26.4 (2.2)/  26.4 (2.3) | 43.6/30.2 |
| Bergmans et al [37], 2023 | Germany and United Kingdom | Postgraduate | Clinic | RCT | Final | Block | 46/44 | N/A | N/A |
| Bhai and Poustinchian [38], 2021 | United States | Undergraduate | Clinic | Non-RCT | Final | Semester | 204/201 | 24 (1.14)/  23.5 (1.3) | 53/53 |
| Bhavsar et al [39], 2022 | India | Undergraduate | Basic | Non-RCT | Final | Block | 39/43 | N/A | N/A |
| Bhide et al [40], 2022 | India | Undergraduate | Basic | Non-RCT | Final | Block | 175/175 | N/A | N/A |
| Homsanit [41], 2019 | Thailand | Undergraduate | Public health | Non-RCT | Final | Block | 313/315 | 19.5 (0.6)/  19.6 (0.9) | 48.6/48.6 |
| Bouwmeester et al [42], 2019 | Netherland | Undergraduate | Clinic | Non-RCT | Final | Semester | 36/28 | 23.1 (1.1)/  24.1 (3.6) | 67/64 |
| Boysen-Osborn et al [43], 2016 | United States | Undergraduate | Clinic | Non-RCT | Final | Block | 95/259 | N/A | N/A |
| Burak et al [44], 2017 | Canada | Undergraduate | Clinic | Non-RCT | Final | Block | 158/163 | N/A | N/A |
| Cai et al [45], 2022 | China | Undergraduate | Basic | Non-RCT | Final | Block | 59/58 | 20.2 (0.1)/  20.1 (0.1) | 48/45 |
| Cao et al [46], 2023 | China | Undergraduate | Clinic | Non-RCT | Final | Semester | 66/70 | 21.5 (0.75)/ 21.68 (0.53) | 43.9/48.6 |
| Carrick et al [47], 2017 | United States | Postgraduate | Clinic | RCT | Baseline and final | Block | 137/137 | 38.5 (10) for all participants | 26.3/26.3 |
| Cheng et al [48], 2017 | China | Undergraduate | Basic | Non-RCT | Baseline and final | Semester | 24/87 | 18.9 (0.6)/  18.9 (1.0) | 58.3/62.1 |
| Chick et al [49], 2021 | United States | Postgraduate | N/A | Non-RCT | Final | Semester | N/A | N/A | N/A |
| Chiu et al [50], 2018 | Taiwan | Undergraduate | Clinic | Non-RCT | Final | Semester | 30/29 | N/A | 26.7/37.9 |
| Chowdhury et al [51], 2019 | United Kingdom | Undergraduate | Clinic | Non-RCT | Final | Semester | 310/234 | N/A | N/A |
| Ding et al [52], 2019 | China | Undergraduate | Clinic | Non-RCT | Baseline and final | Semester | 32/35 | 22.8 (0.9)/  22.9 (0.9) | 65.6/62.9 |
| El Sadik and Al Abdulmonem [53], 2021 | Saudi Arabia | Undergraduate | Basic | Non-RCT | Final | Block | 46/49 | 19.1 (0.4)/  19.1 (0.5) | 100/100 |
| El-Ashkar et al [54], 2022 | Saudi Arabia | Undergraduate | Basic | Non-RCT | Final | Semester | 81/84 | N/A | N/A |
| Evans et al [55], 2016 | United States | Undergraduate | Basic | Non-RCT | Final | Semester | 101/178 | N/A | N/A |
| Fakhoury et al [56], 2021 | Saudi Arabia | Undergraduate | Basic | Non-RCT | Final | Semester | 247/247 | N/A | 61/61 |
| Feng et al [57], 2022 | China | Undergraduate | Basic | Non-RCT | Final | Semester | 31/31 | 21.06 (0.77)/ 21.19 (0.75) | 61.3/67.7 |
| Galway et al [58], 2014 | Canada | Postgraduate | Public health | Non-RCT | Final | Semester | 11/22 | N/A | N/A |
| Gillispie [59], 2016 | United States | Undergraduate | Clinic | Non-RCT | Final | Semester | 22/14 | N/A | 40.9/28.6 |
| Gong et al [60], 2021 | China | Undergraduate | Clinic | Non-RCT | Final | N/A | 100/100 | 22 (0.4)/  22 (0.4) | 50/49 |
| Graham et al [61], 2019 | United States | Postgraduate | Clinic | Non-RCT | Baseline and final | Block | 59/59 | 27.3 (2.2)/  26.6 (6.2) | 38/40 |
| Granero Lucchetti et al [62], 2018 | Brazil | Undergraduate | Public health | Non-RCT | Baseline and final | Semester | 83/83 | N/A | N/A |
| Grant et al [63], 2021 | United States | Undergraduate | Clinic | Non-RCT | Final | Block | 113/257 | N/A | N/A |
| Gutierrez-Gonzalez et al [64], 2023 | Spain | Undergraduate | Clinic | Non-RCT | Final | Block | 69/187 | N/A | N/A |
| Heitmann et al [65], 2023 | Germany | Undergraduate | Clinic | RCT | Baseline and final | Semester | 82/92 | N/A | N/A |
| Heitz et al [66], 2015 | United States | Undergraduate | Clinic | RCT | Final | Block | 56/56 | N/A | N/A |
| Hernandez-Guerra et al [67], 2021 | Spain | Undergraduate | Clinic | Non-RCT | Final | Block | 404/404 | N/A | N/A |
| Herrero and Quiroga [68], 2020 | Spain | Undergraduate | Basic | Non-RCT | Baseline and final | Semester | 201/229 | N/A | 61/68 |
| Hsu et al [69], 2016 | Taiwan | Postgraduate | Clinic | Non-RCT | Final | Semester | 39/43 | 26.9 (0.9)/ 26.5 (1.1) | 35.9/34.9 |
| Hu et al [70], 2019 | China | Undergraduate | Clinic | RCT | Baseline and final | Block | 37/37 | 22.1 (1)/  22.4 (0.9) | 51.4/48.6 |
| Hu et al [71], 2024 | China | Undergraduate | Clinic | RCT | Final | Semester | 46/46 | 21.15 (0.92)/  20.85 (0.92) | 58.7/52.2 |
| Huang et al [72], 2020 | Taiwan | Undergraduate | Basic | Non-RCT | Final | Block | 38/24 | 20.3 (1.5)/ 20.4 (1.3) | 86.8/83.3 |
| Ilic et al [73], 2015 | Australia | Undergraduate | Basic | RCT | Final | N/A | 73/74 | N/A | N/A |
| Ito et al [74], 2022 | Japan | Undergraduate | Clinic | Non-RCT | Final | Block | 107/62 | N/A | N/A |
| Jalali et al [75], 2020 | Canada | Undergraduate | Basic | Non-RCT | Final | Block | N/A | N/A | N/A |
| Ji et al [76], 2022 | China | Undergraduate | Basic | Non-RCT | Final | Semester | 58/61 | 19.7 (0.69)/ 19.8 (0.72) | 48.3/52.5 |
| Jumaa et al [77], 2024 | Saudi Arabia | Undergraduate | Basic | RCT | Baseline and final | Semester | 198/198 | N/A | 30.3/30.3 |
| Jung et al [78], 2018 | Korea | Undergraduate | Clinic | Non-RCT | Final | N/A | 40/40 | N/A | 40/40 |
| Kasat et al [79], 2023 | India | Undergraduate | Basic | Non-RCT | Final | Block | 50/50 | N/A | 34/34 |
| Ketterer et al [80], 2021 | United States | Postgraduate | Clinic | Non-RCT | Final | N/A | 33/31 | N/A | N/A |
| Khojasteh et al [81], 2021 | Iran | Undergraduate | Basic | Non-RCT | Baseline and final | Semester | 47/47 | N/A | N/A |
| King et al [82], 2018 | United States | Postgraduate | Clinic | Non-RCT | Final | Semester | 31/36 | N/A | N/A |
| Kiviniemi [83], 2014 | United States | Postgraduate | Public health | Non-RCT | Final | Semester | 38/28 | N/A | N/A |
| Kolahdouzan et al [84], 2020 | Iran | Undergraduate | Clinic | RCT | Final | Block | 25/25 | N/A | 60/56 |
| Kong et al [85], 2020 | China | Undergraduate | Basic | Non-RCT | Final | Semester | 125/123 | N/A | N/A |
| Kuhl et al [86], 2017 | Germany | Undergraduate | Basic | Non-RCT | Final | Semester | 40/299 | N/A | N/A |
| Langdorf et al [87], 2018 | United States | Undergraduate | Clinic | Non-RCT | Final | Block | 209/259 | N/A | N/A |
| Lewis et al [88], 2018 | United States | Undergraduate | N/A | Non-RCT | Final | Semester | 98/102 | N/A | N/A |
| Li et al [89], 2023 | China | Postgraduate | Clinic | RCT | Final | Block | 39/39 | 25.21 (1.17)/ 25.44 (1.1) | 53.8/56.4 |
| Li et al [90], 2023 | China | Postgraduate | Public Health | Non-RCT | Final | Semester | 21/22 | N/A | 71.4/50 |
| Liao et al [91], 2023 | China | Undergraduate | Clinic | RCT | Final | Semester | 48/48 | 21.77 (0.77)/ 21.52 (0.64) | 58.3/58.3 |
| Liebert et al [92], 2016 | United States | Undergraduate | Clinic | Non-RCT | Final | Semester | 89/92 | N/A | N/A |
| Lin et al [93], 2017 | China | Undergraduate | Clinic | RCT | Baseline and final | N/A | 22/22 | 23.5 (1.1)/ 24.2 (2.2) | 59/55 |
| Liu et al [94], 2021 | China | Undergraduate | N/A | Non-RCT | Final | N/A | 61/58 | 22.83 (1.24)/ 22.94 (1.35) | 9.8/8.6 |
| Liu et al [95], 2024 | China | Postgraduate | Clinic | Non-RCT | Baseline and final | Semester | 37/39 | 22.49 (1.33)/ 22.21 (1.13) | 32.4/38.5 |
| Liu et al [96], 2024 | China | Undergraduate | Clinic | Non-RCT | Baseline and final | Semester | 49/50 | 20.84 (0.11)/ 20.9 (0.1) | 71.4/68 |
| Lopinska et al [97], 2022 | Poland | Undergraduate | Basic | Non-RCT | Final | Semester | 210/453 | N/A | 52.2/46.9 |
| Lu et al [98], 2021 | United States | Postgraduate | Clinic | RCT | Baseline and final | Block | 110/103 | N/A | N/A |
| Lu et al [99], 2023 | China | Undergraduate | Basic | RCT | Final | Semester | 62/69 | N/A | N/A |
| Lucardie and Busari [100], 2017 | Netherland | Postgraduate | Clinic | Non-RCT | Baseline and final | Block | 8/10 | 29 (2.25)/ 28.64 (2.5) | 70/58.3 |
| Ma et al [101], 2018 | China | Undergraduate | Basic | RCT | Baseline and final | Semester | 42/50 | 19.4 (0.74)/ 19.5 (0.81) | 50/56 |
| Malhotra and Bhagat [102], 2023 | India | Undergraduate | Basic | Non-RCT | Final | Block | 95/95 | N/A | N/A |
| Malik et al [103], 2020 | Pakistan | Postgraduate | N/A | RCT | Baseline and final | Block | 40/40 | N/A | N/A |
| Marchalot et al [104], 2018 | France | Postgraduate | Basic | Non-RCT | Final | N/A | 54/95 | N/A | N/A |
| Marshall and Conroy [105], 2022 | United States | Undergraduate | Clinic | Non-RCT | Final | Block | 162/170 | 23.6 (1.33)/ 23.5 (1.33) | 50.6/50.6 |
| Martinelli et al [106], 2017 | United States | Postgraduate | Clinic | Non-RCT | Baseline and final | Block | 81/56 | 28.7 (2.5)/ 28.8 (2.6) | 33.3/44.6 |
| Mengesha et al [107], 2024 | Ethiopia | Undergraduate | Basic | RCT | Baseline and final | Semester | 50/50 | N/A | N/A |
| Moll-Khosrawi et al [108], 2021 | Germany | Undergraduate | Clinic | RCT | Baseline and final | Semester | 35/45 | 22.8 (1.24)/ 22.9 (1.35) | 48.6/53.3 |
| Moraros et al [109], 2015 | Canada | Postgraduate | Public health | Non-RCT | N/A | N/A | 60/52 | N/A | N/A |
| Morton and Colbert-Getz [110], 2017 | United States | Undergraduate | Basic | Non-RCT | Final | Semester | 102/101 | N/A | 48/52.5 |
| Moskowitz and Hsueh [111], 2020 | United States | Postgraduate | Clinic | Non-RCT | N/A | N/A | N/A | N/A | N/A |
| Nanjundaiah and Anuradha [112], 2024 | India | Undergraduate | Basic | Non-RCT | Final | Block | 50/50 | N/A | N/A |
| Newman et al [113], 2021 | United States | Undergraduate | Clinic | Non-RCT | N/A | N/A | 170/168 | N/A | N/A |
| Nourinezhad et al [114], 2021 | Iran | Undergraduate | Basic | Non-RCT | Baseline and final | Semester | 25/25 | N/A | N/A |
| O’Connor et al [115], 2016 | United States | Undergraduate | Clinic | Non-RCT | Baseline and final | Block | 72/103 | N/A | N/A |
| Ohlenburg et al [116], 2024 | Germany | Undergraduate | Clinic | RCT | Final | Semester | 52/51 | 25.44 (3.55)/ 25.5 (4.14) | 69.2/66.7 |
| Paralikar et al [117], 2022 | India | Undergraduate | Basic | Non-RCT | Baseline and final | Block | 102/102 | N/A | N/A |
| Peterson et al [118], 2017 | United States | Postgraduate | Clinic | Non-RCT | Final | N/A | 9/19 | N/A | N/A |
| Pontius et al [119], 2020 | United States | Postgraduate | N/A | Non-RCT | Final | N/A | N/A | N/A | N/A |
| Porcaro et al [120], 2016 | Australia | Undergraduate | Clinic | Non-RCT | Final | N/A | 68/95 | N/A | N/A |
| Prabhavathi et al [121], 2024 | India | Undergraduate | Basic | RCT | Final | Semester | 75/75 | N/A | 56/56 |
| Prabhu and Prabhu [122], 2022 | Malaysia | Undergraduate | Basic | Non-RCT | Baseline and final | Block | 21/21 | N/A | N/A |
| Qian et al [123], 2021 | China | Undergraduate | N/A | RCT | Baseline and final | N/A | 37/37 | N/A | N/A |
| Qutob [124], 2022 | Saudi Arabia | Undergraduate | Clinic | Non-RCT | Final | Semester | 24/30 | N/A | 83.3/90 |
| Rathner and Schier [125], 2020 | Australia | Undergraduate | Clinic | Non-RCT | Final | Semester | 138/132 | N/A | N/A |
| Riddell et al [126], 2017 | United States | Postgraduate | Clinic | Non-RCT | Baseline and final | N/A | 38/37 | N/A | N/A |
| Rui et al [127], 2017 | China | Undergraduate | Clinic | RCT | Final | Block | 90/91 | 20.84 (0.67)/ 20.9 (0.58) | 54.4/45.1 |
| Sajid et al [128], 2016 | Saudi Arabia | Undergraduate | Clinic | Non-RCT | Final | N/A | 154/155 | N/A | N/A |
| Sanchez et al [129], 2020 | Colombia | Undergraduate | Basic | Non-RCT | Final | N/A | 29/46 | 21 (3)/23 (3) | 55/52 |
| Seidi et al [130], 2024 | Iran | Undergraduate | Basic | RCT | Baseline and final | Semester | 30/30 | 20.76 (1.92)/ 21.36 (2.3) | 53.3/56.7 |
| Sezer and Abay [131], 2018 | Turkey | Undergraduate | Clinic | RCT | Baseline and final | Semester | 19/19 | N/A | 36.8/36.8 |
| Sezer and Elcin [132], 2020 | Turkey | Undergraduate | Clinic | RCT | Final | Semester | 183/180 | N/A | N/A |
| Shabani et al [133], 2020 | Iran | Undergraduate | Clinic | Non-RCT | Baseline and final | Block | 30/29 | N/A | N/A |
| Shahid et al [134], 2024 | Pakistan | Undergraduate | Basic | RCT | Baseline and final | Semester | 67/63 | 20.37 (0.11)/ 20.55 (0.11) | N/A |
| Shiau et al [135], 2018 | United States | Postgraduate | Public health | Non-RCT | Final | N/A | 78/71 | N/A | N/A |
| Shoemaker et al [136], 2022 | United States | Undergraduate | N/A | Non-RCT | Baseline and final | Semester | N/A | N/A | N/A |
| Smith and Boscak [137], 2021 | United States | N/A | N/A | Non-RCT | N/A | N/A | N/A | N/A | N/A |
| Sourg et al [138], 2023 | Sudan | Undergraduate | Clinic | RCT | Baseline and final | Block | 30/33 | N/A | 40/54.5 |
| Street et al [139], 2014 | United States | Undergraduate | Basic | Non-RCT | Final | Block | 177/180 | N/A | 52.8/45 |
| Tahir et al [140], 2020 | Saudi Arabia | Undergraduate | Clinic | Non-RCT | Final | Semester | 136/136 | N/A | 100/100 |
| Tang et al [141], 2017 | China | Undergraduate | Clinic | RCT | Baseline and final | Block | 48/47 | 22.3 (0.6)/  22.6 (0.4) | 47.9/44.7 |
| Teichgraber et al [142], 2021 | Germany | Undergraduate | Clinic | Non-RCT | N/A | Semester | 266/750 | N/A | N/A |
| Nassiri Toosi et al [143], 2021 | Iran | Undergraduate | Clinic | Non-RCT | Final | N/A | 97/101 | 20.6 (1.2)/  20.7 (1.1) | 52.6/52.5 |
| Tsao et al [144], 2022 | Taiwan | Undergraduate | Clinic | Non-RCT | Final | Semester | 45/68 | 23.2 (3.17)/ 23.85 (1.09) | 37.8/42.6 |
| Tune et al [145], 2013 | United States | Postgraduate | Basic | Non-RCT | Final | Semester | 13/14 | N/A | 76.9/14.3 |
| Tusa et al [146], 2018 | Finland | Undergraduate | Clinic | Non-RCT | Final | Semester | 39/40 | N/A | 46.2/50 |
| Uchida et al [147], 2022 | Japan | Undergraduate | Clinic | Non-RCT | Baseline and final | Semester | 39/44 | 23.2 (2.5)/  23 (1.4) | 20.5/18.2 |
| Veeramani et al [148], 2015 | India | Undergraduate | Clinic | Non-RCT | N/A | N/A | N/A | N/A | N/A |
| Wang et al [149], 2020 | China | Undergraduate | Public health | Non-RCT | Baseline and final | Semester | 44/44 | N/A | N/A |
| Wang et al [150], 2021 | China | Postgraduate | Clinic | RCT | Final | N/A | 55/55 | N/A | N/A |
| Wang et al [151], 2022 | China | Postgraduate | Clinic | RCT | Baseline and final | Block | 36/39 | 24.17 (0.5)/ 23.9 (0.5) | 80.6/71.8 |
| Weiwei et al [152], 2019 | China | Undergraduate | Basic | Non-RCT | Final | N/A | 88/92 | N/A | N/A |
| Yang et al [153], 2020 | China | Undergraduate | Basic | RCT | Baseline and final | Semester | 45/44 | 19.71 (0.75)/ 19.74 (0.74) | 88.9/88.6 |
| Yang et al [154], 2021 | China | Undergraduate | Clinic | Non-RCT | Final | N/A | 31/31 | N/A | 38.7/32.3 |
| Zhang et al [155], 2019 | China | Undergraduate | Basic | RCT | Baseline and final | N/A | 115/82 | N/A | N/A |
| Zhang et al [156], 2022 | China | Postgraduate | Clinic | RCT | Final | N/A | 42/37 | 27.57 (3.42)/ 27.62 (2.95) | 73.8/75.7 |
| Zhang et al [157], 2024 | China | Undergraduate | Clinic | RCT | Final | Block | 29/35 | 27.66 (2.99)/ 27.6 (3.35) | 72.4/74.3 |
| Zhang et al [158], 2024 | China | Postgraduate | Clinic | RCT | Final | Block | 45/45 | 24.37 (2.59)/ 25.4 (2.78) | 48.9/53.3 |
| Zheng et al [159], 2022 | China | Undergraduate | Clinic | RCT | Final | Block | 51/52 | 24.55 (1.14)/ 24.52 (1) | 51/42.3 |
| Zhong et al [160], 2022 | China | Undergraduate | Basic | Non-RCT | Final | Semester | 93/89 | N/A | N/A |
| Zhou et al [161], 2024 | China | Undergraduate | Clinic | Non-RCT | Final | Semester | 64/58 | N/A | N/A |

^a^FC: flipped classroom. ^b^C: control. ^c^N/A: not available.


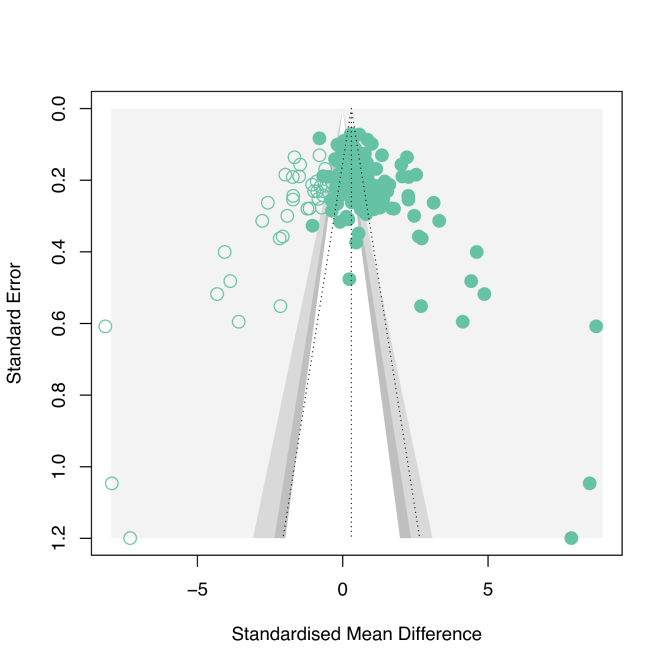
**
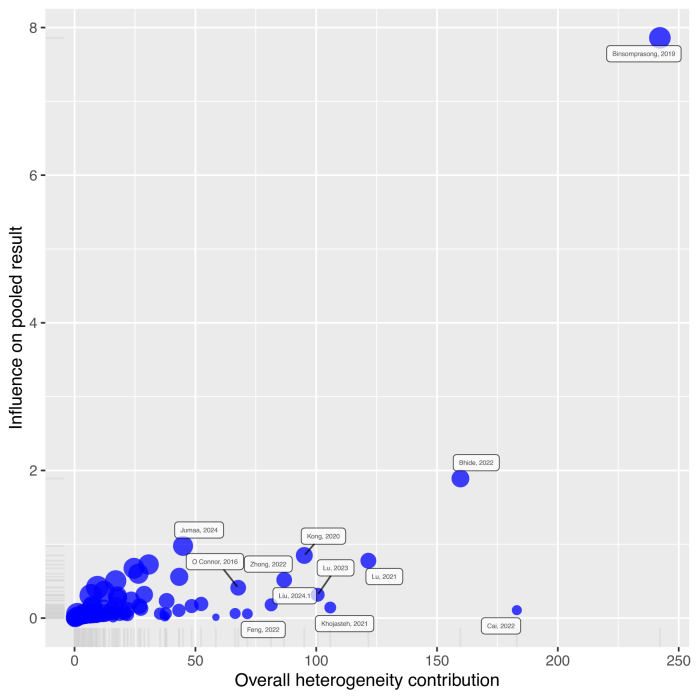
**

**Figure S1a)** Baujat graph for final knowledge test score **b)** Funnel plot - Publication bias for final knowledge score

**
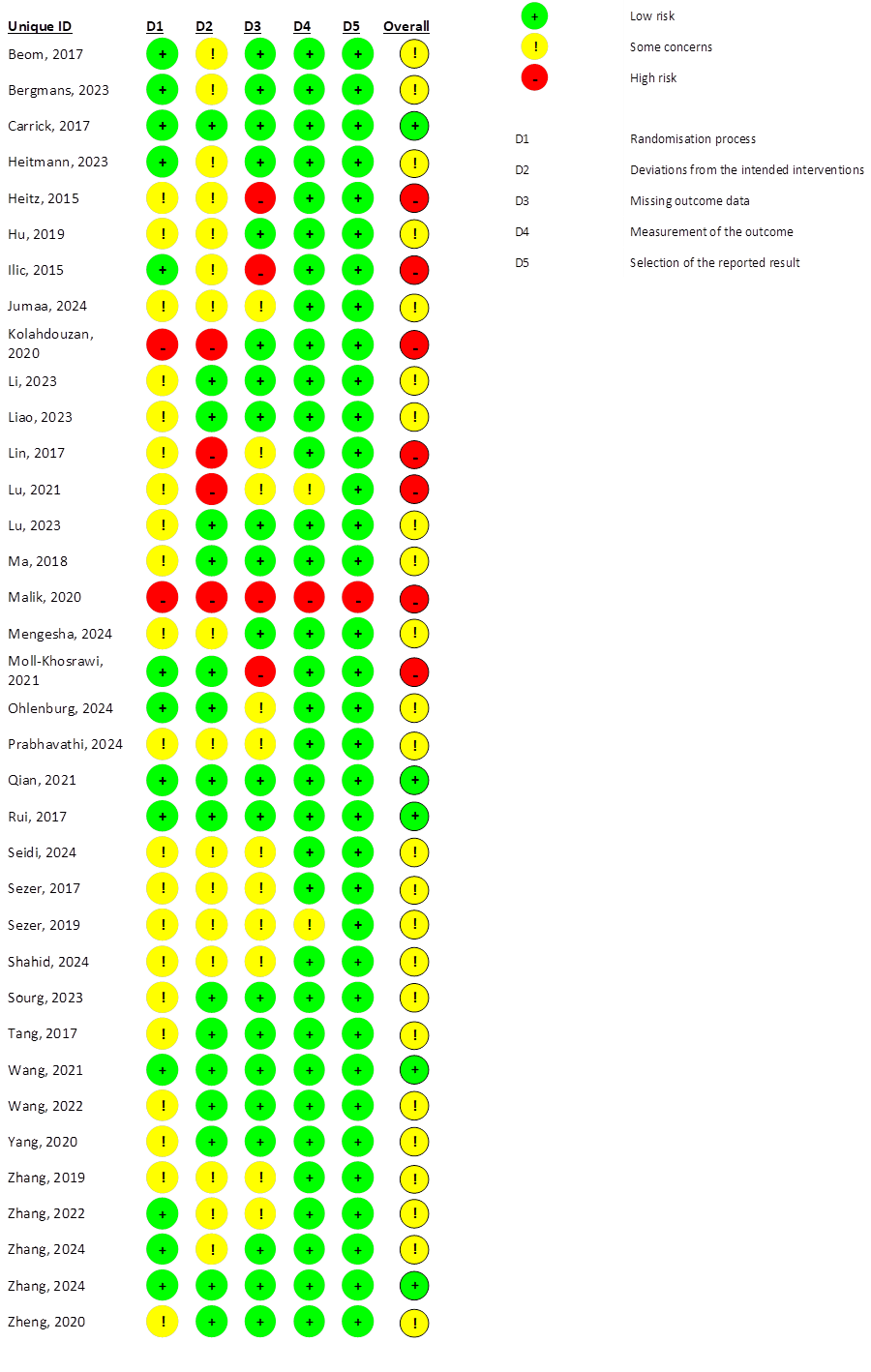
**

**Figure S2** Assessment of the risk of bias of RCT studies

**
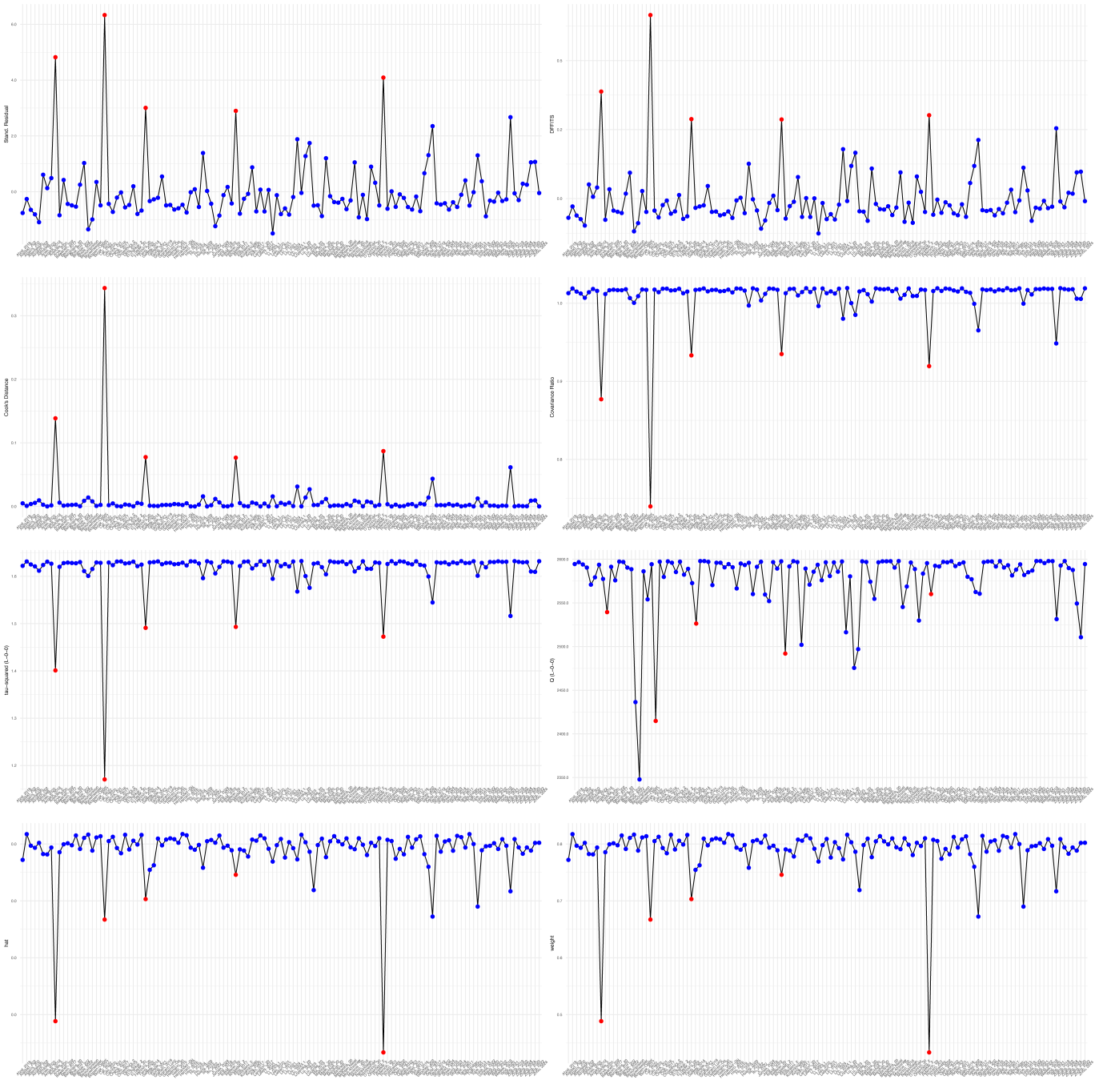
**

**Figure S3** Influence analysis for final knowledge score

**Table S2.** Subgroup analysis of studies reporting knowledge scores

| **Subgroup Analysis** | **n** | **SMD (95% CI)** | **I^2^** |
| --- | --- | --- | --- |
| Observational | 90 | 0.90 (0.59; 1.20] | 95.6% |
| RCT | 37 | 0.93 (0.65; 1.22) | 93.2% |
| Undergraduate | 101 | 0.90 (0.64; 1.16) | 95.5% |
| Postgraduate | 26 | 0.88 (0.44; 1.32) | 93.2% |
| Clinic | 72 | 0.71 (0.51; 0.91) | 91.8% |
| Public Health | 9 | 0.44 (-0.24; 1.12) | 96.6% |
| Basic | 41 | 1.38 (0.80; 1.96) | 96.9% |
| Block | 47 | 0.90 (0.54; 1.27) | 96.2% |
| Semester | 58 | 0.88 (0.54; 1.23) | 94.8% |
| Not same instructors | 13 | 1.11 (0.41; 1.81) | 96.5% |
| Same instructors | 28 | 1.22 [0.55; 1.88) | 96.5% |

**
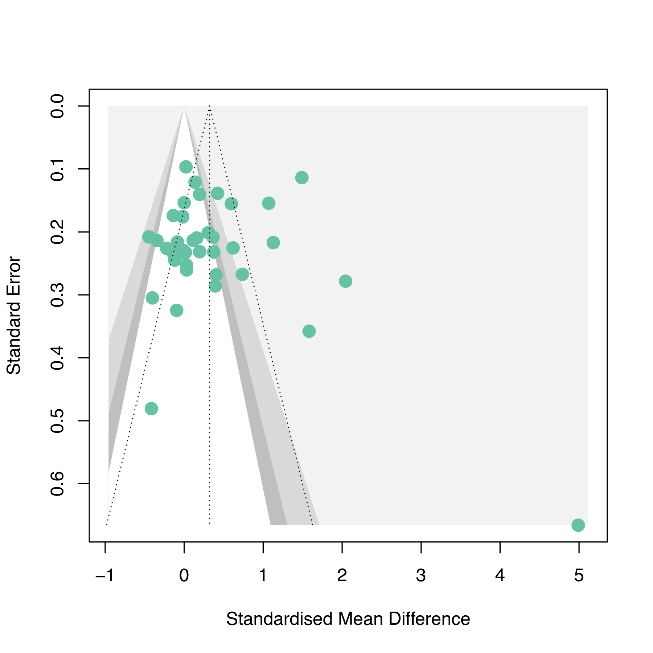

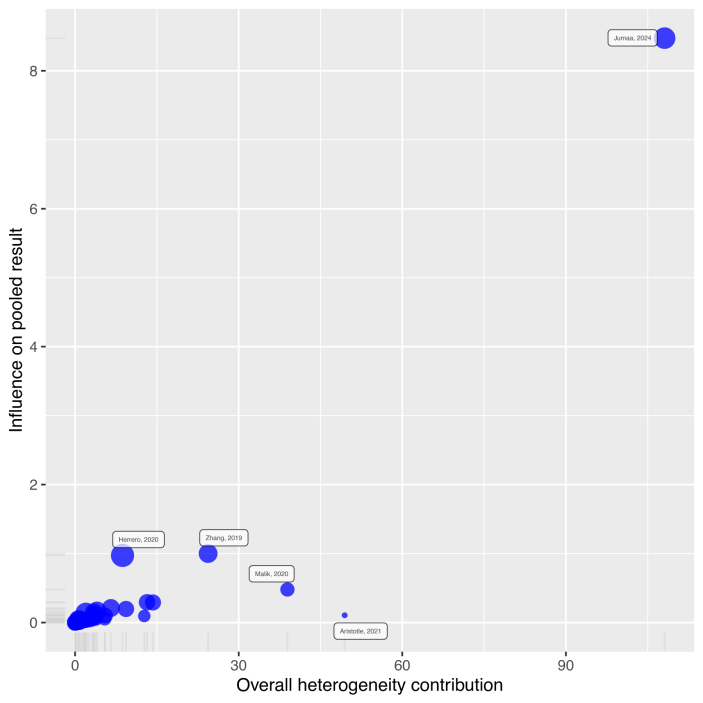
**

**
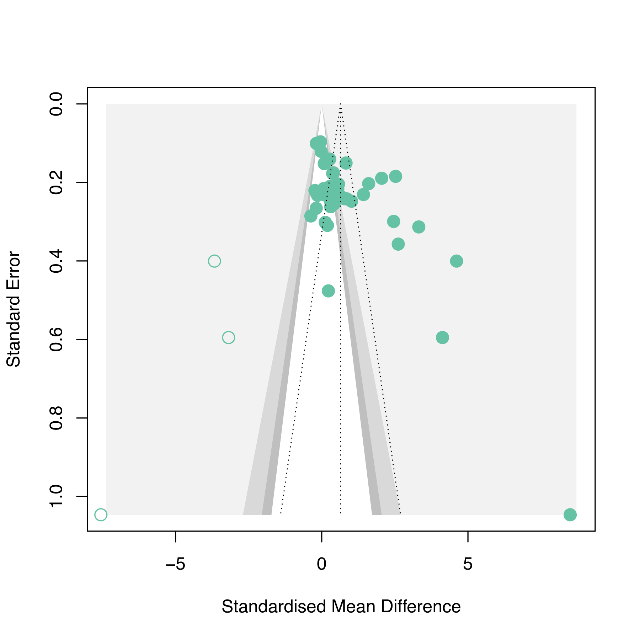

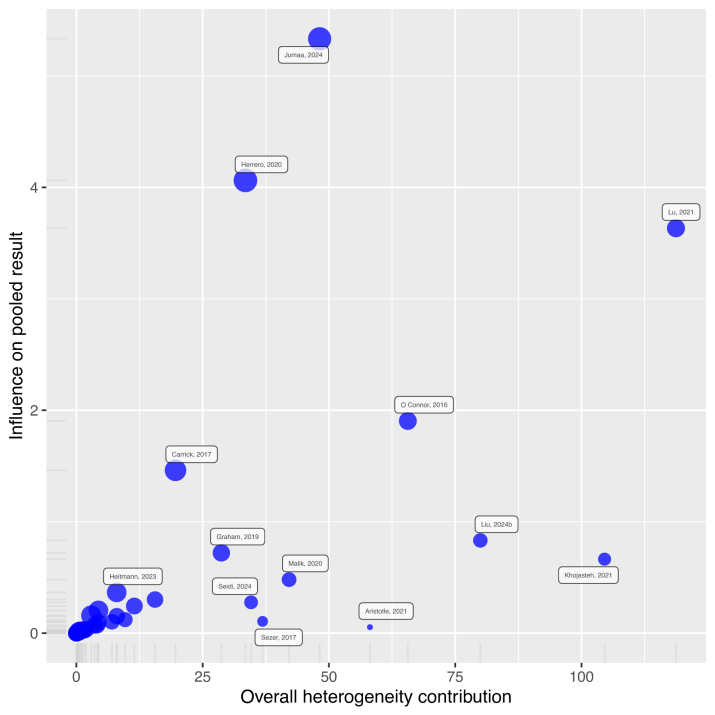
**

**Figure S4 a**) Baujat graph for baseline knowledge score **b)** Baujat graph for final knowledge score in studies reporting baseline knowledge assessment **c)** Funnel plot - Publication bias for baseline knowledge score **d)** Funnel plot - Publication bias for final knowledge score in studies reporting baseline knowledge assessment


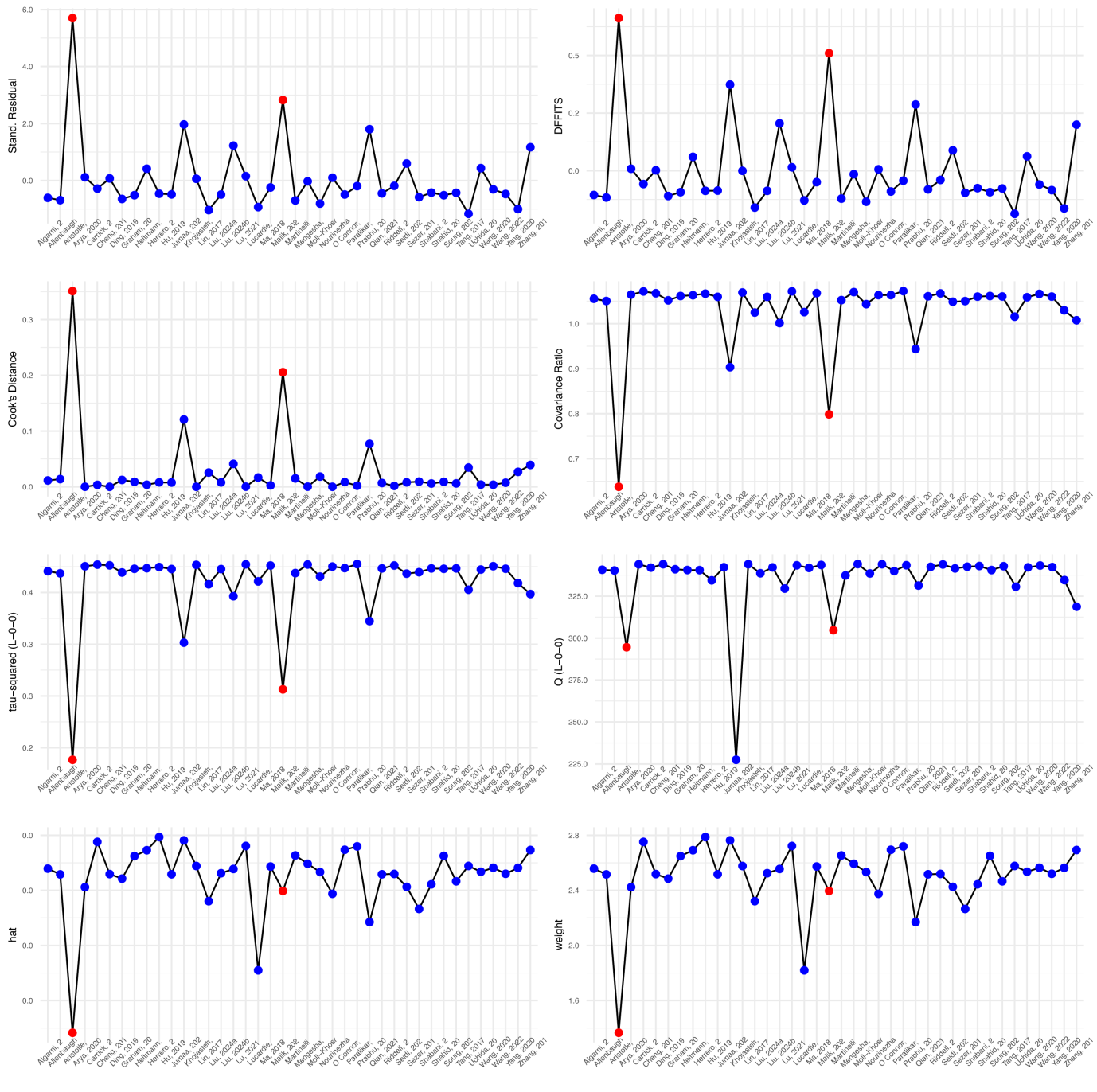


**Figure S5** Influence analysis for baseline knowledge score


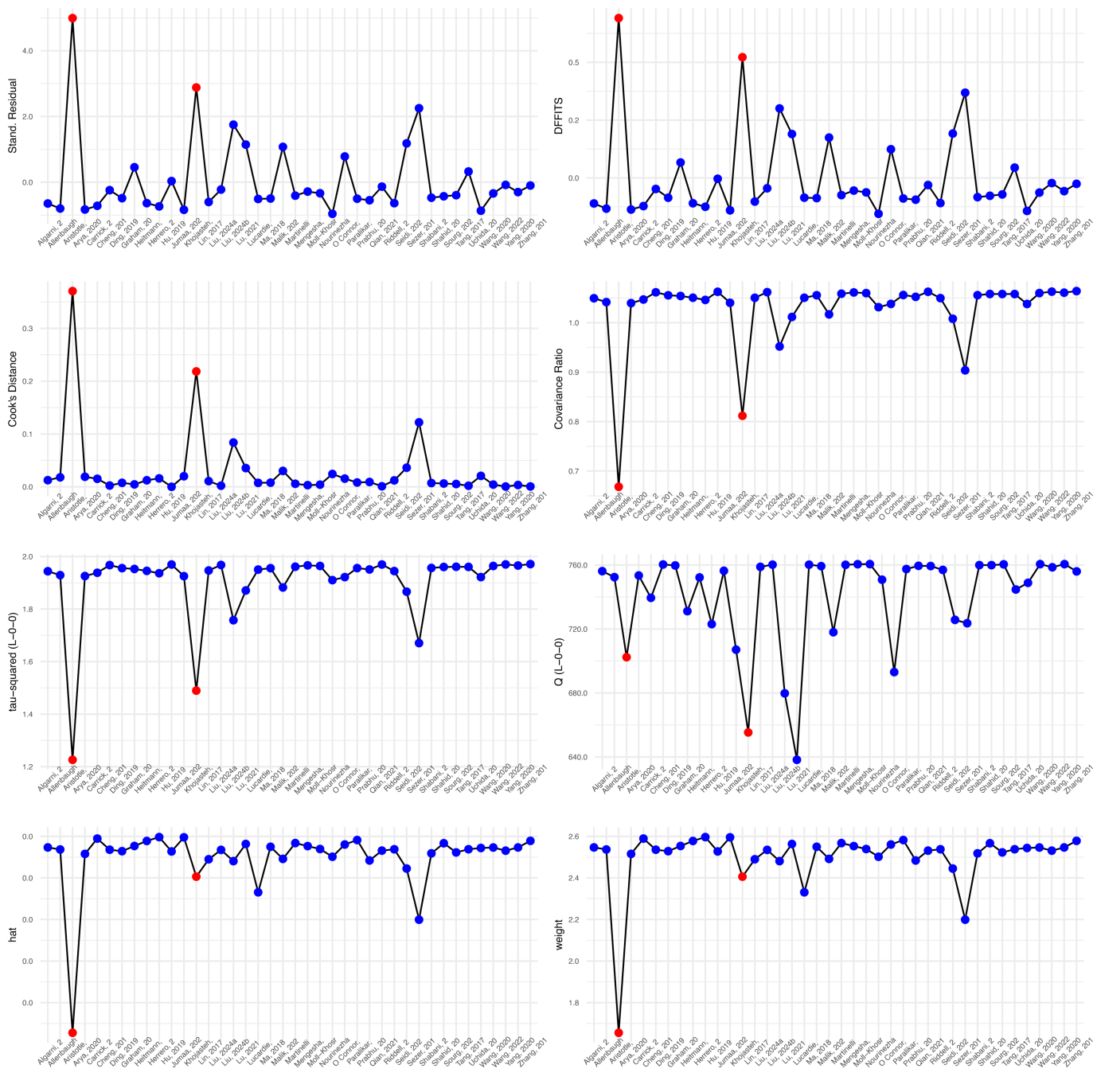


**Figure S6** Influence analysis for final knowledge score in studies reporting baseline knowledge assessment

**
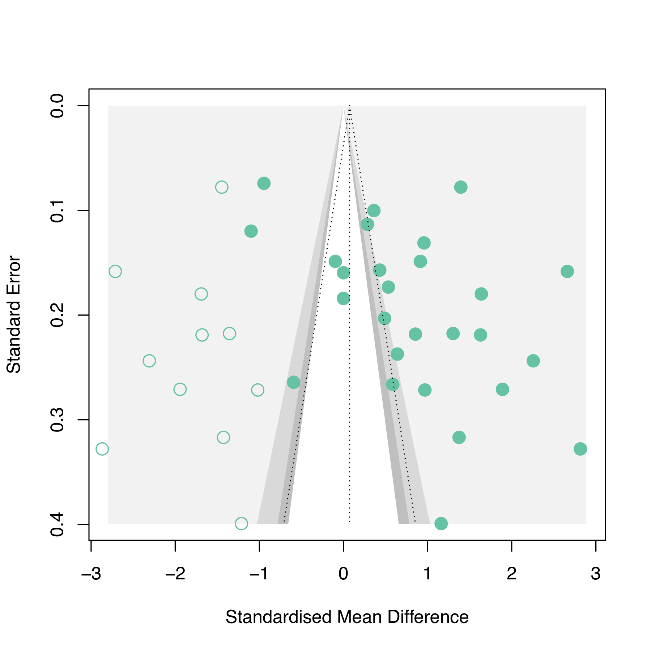

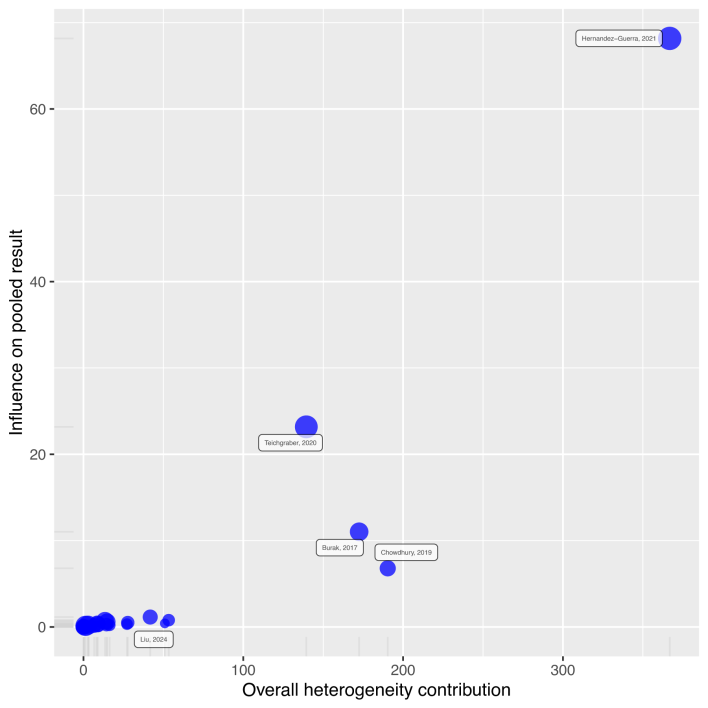
**

**Figure S7 a)** Baujat graph for students’ satisfaction score **b)** Funnel plot - Publication bias for students’ satisfaction score


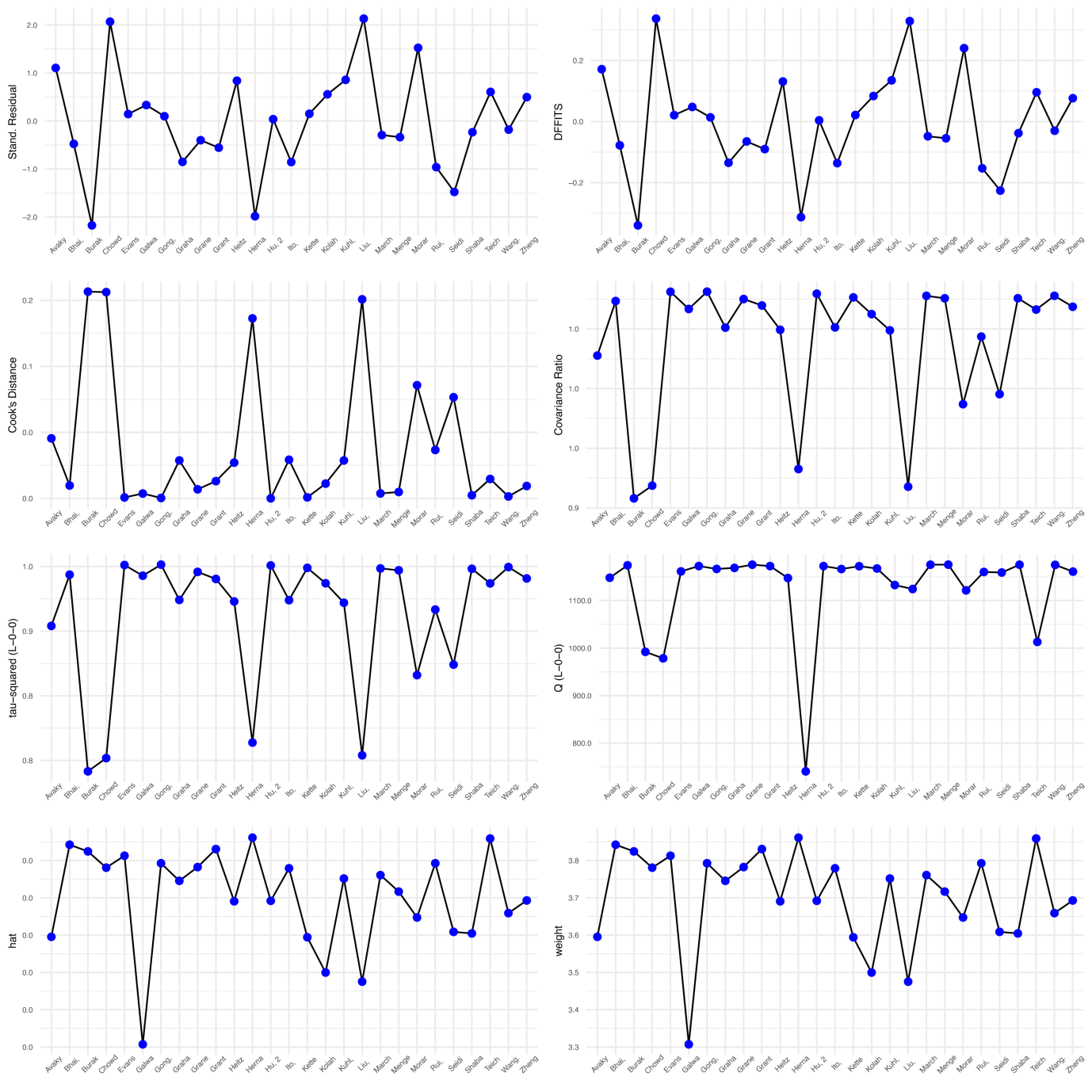


**Figure S8** Influence analysis for students’ satisfaction score


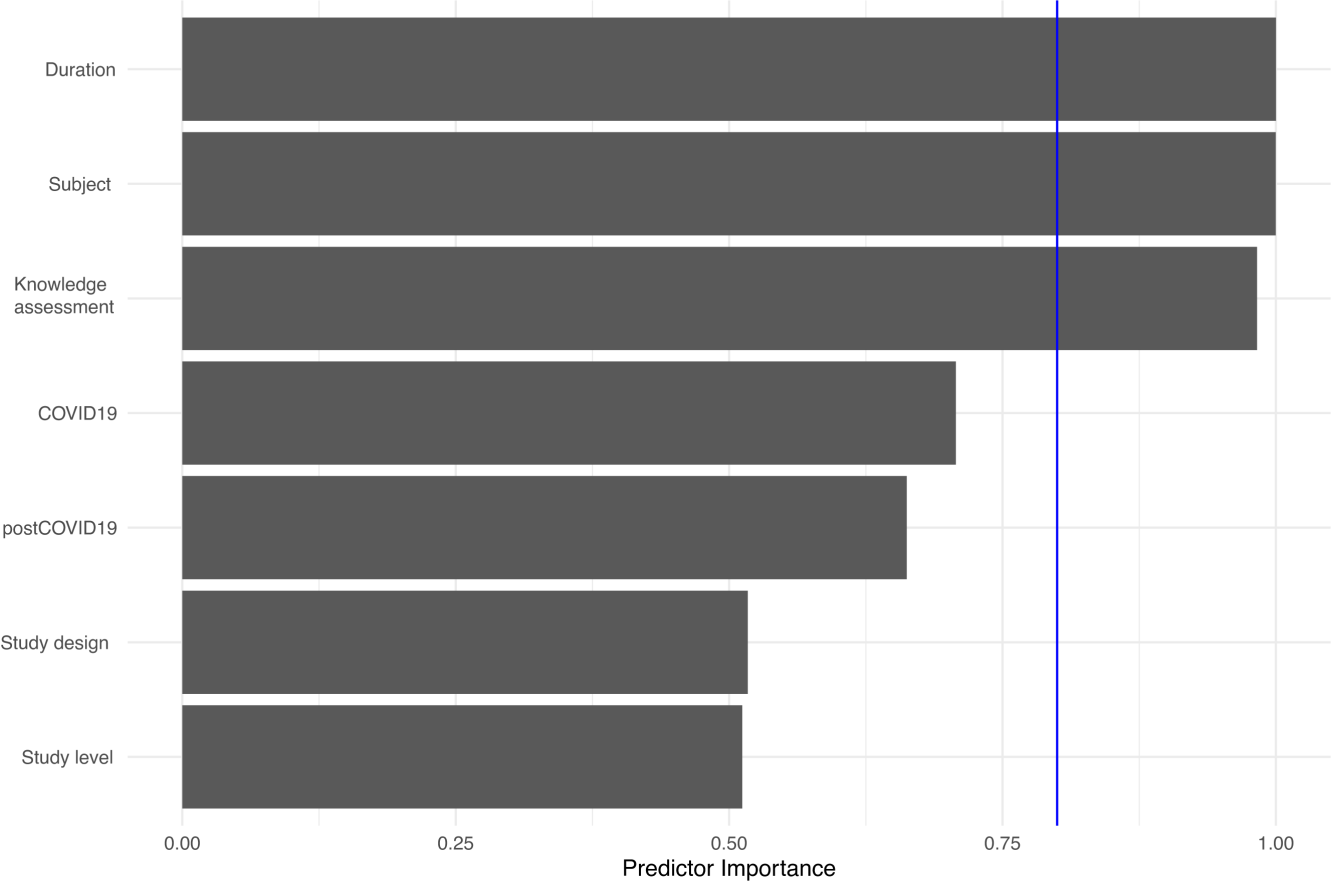


**Figure S9** Predictor importance analysis for final knowledge scores as outcome measure
